# Genome sequencing reveals a new lineage associated with lablab bean and genetic exchange between *Xanthomonas axonopodis* pv. *phaseoli* and *Xanthomonas fuscans* subsp. *fuscans*

**DOI:** 10.3389/fmicb.2015.01080

**Published:** 2015-10-07

**Authors:** Valente Aritua, James Harrison, Melanie Sapp, Robin Buruchara, Julian Smith, David J. Studholme

**Affiliations:** ^1^International Center for Tropical AgricultureKampala, Uganda; ^2^Biosciences, University of ExeterExeter, UK; ^3^Fera Science Ltd.York, UK; ^4^Africa Regional Office, International Center for Tropical Agriculture, Consultative Group for International Agricultural Research (CGIAR)Nairobi, Kenya

**Keywords:** beans, *Phaseolus vulgaris*, *Phaseolus lunatus*, *Lablab purpureus*, *Dolichos lablab*, *Xanthomonas fuscans*, *Xanthomonas axonopodis*

## Abstract

Common bacterial blight is a devastating seed-borne disease of common beans that also occurs on other legume species including lablab and Lima beans. We sequenced and analyzed the genomes of 26 strains of *Xanthomonas axonopodis* pv. *phaseoli* and *X. fuscans* subsp. *fuscans*, the causative agents of this disease, collected over four decades and six continents. This revealed considerable genetic variation within both taxa, encompassing both single-nucleotide variants and differences in gene content, that could be exploited for tracking pathogen spread. The bacterial strain from Lima bean fell within the previously described Genetic Lineage 1, along with the pathovar type strain (NCPPB 3035). The strains from lablab represent a new, previously unknown genetic lineage closely related to strains of *X. axonopodis* pv. *glycines*. Finally, we identified more than 100 genes that appear to have been recently acquired by *Xanthomonas axonopodis* pv. *phaseoli* from *X. fuscans* subsp. *fuscans*.

## Introduction

Common bacterial blight (CBB) is a devastating, widespread and seed-borne disease of common beans (*Phaseolus vulgaris*). The bacteria that cause CBB are genetically diverse (Gilbertson et al., 1991; Alavi et al., 2008; Parkinson et al., 2009; Fourie and Herselman, 2011) and include fuscous strains, which produce a brown pigment on tyrosine-containing medium, and non-fuscous strains. Currently, the non-fuscous strains are classified as *Xanthomonas axonopodis* pv. *phaseoli* (*Xap*) while the fuscous strains are classified into a different species as *X. fuscans* subsp. *fuscans* (*Xff*) (Schaad et al., 2005; Bull et al., 2012), though some authors consider the species *X. fuscans* to be a subclade within *X. axonopodis* (Rodriguez-R et al., 2012; Mhedbi-Hajri et al., 2013).

The main host of *Xap* and *Xff* is common bean (*Phaseolus vulgaris*) but they have also been isolated from the closely related Lima bean (*Phaseolus lunatus*) and lablab bean (*Lablab purpureus*, formerly *Dolichos lablab*) as well as several other legumes, including *Vigna* species (Bradbury, 1986). Lablab bean is a drought-resistant legume that stays green during the dry season and is used to improve soil and to feed livestock (Schaaffhausen, 1963). Lablab bean has been reported to be the main leguminous fodder crop used in Sudan around Khartoum, where it is known as hyacinth bean, bonavist bean or, in Arabic, lubia afin (Schaaffhausen, 1963). It is also grown in Sudan as a pulse legume (Mahdi and Atabani, 1992). Infection by *Xap* has been observed when lablab was sown during the rainy months (Tarr, 1958). It is not currently clear whether a single bacterial population moves frequently between host species or to what extent CBB agents colonizing different plant species represent distinct and genetically isolated populations or distinct taxa. For example, do the strains from Lima bean and lablab bean belong to the same genetic lineages as do strains from common bean?

A key determinant of pathogenicity in *Xanthomonas* species is the Hrp type-three secretion system (T3SS), which functions as a molecular syringe that secretes and translocates a number of bacterial effector proteins into the cytoplasm of the host cell, thereby modifying the host defenses to the advantage of the pathogen (Alfano and Collmer, 1997, 2004; Galán and Collmer, 1999; Grant et al., 2006; Kay and Bonas, 2009). Host ranges of *X. axonopodis* are significantly associated with the bacteria's repertoires of effectors and phylogenetically distinct strains' convergence on a common host plant might be at least partially explained by shared effectors (Hajri et al., 2009). The particular set of effectors expressed by a pathogen has some practical implications; many plant resistance genes trigger host defenses in response to detection of specific pathogen effectors. Effectors may act as virulence factors, enabling the pathogen to overcome host defenses. Therefore, rational deployment of available genetic resistance depends on knowledge of which effectors are likely to be present in a pathogen population. For example, it might be prudent to deploy resistance genes that recognize core effectors that are present in all strains of the pathogen that the plant will encounter rather than against rarely occurring effectors; this was the rationale for a recent study of the genome sequences of 65 strains of *Xanthomonas axonopodis* pv. *manihotis* (Bart et al., 2012).

CBB is currently a serious challenge to bean production in many African countries. In order to make optimal and rational use of limited available resources to contain and manage the impacts of this disease, it is important to understand the spread pathways of the *Xap* and *Xff* pathogens over both long and short geographical distances. Studies of spread rely on molecular markers that can be used to link strains from different times and locations based on their sharing similar genotypes.

According to multi-locus sequence analysis (MLSA), strains from *Phaseolus* species each fell into one of four genetic lineages (GL): GL 1, GL 2, GL 3, and GL *fuscans* (Mhedbi-Hajri et al., 2013) that corresponded to genetic lineages previously determined on the basis of amplified fragment length polymorphism (AFLP) (Alavi et al., 2008). The MLSA-based genetic lineages are consistent with an earlier classification of *X. axonopodis* strains into “genetic groups” based on conserved repetitive sequences BOX, enterobacterial repetitive intergenic consensus (ERIC), and repetitive extragenic palindromic (REP) (rep-PCR) (Rademaker et al., 2005), though the MLSA-based classification provides higher resolution. Rademaker's genetic group 9.4 includes GL 1, while genetic group 9.6 includes both GL 2 and GL 3 and GL *fuscans* (Mhedbi-Hajri et al., 2013), implying that GL 2 and GL 3 are more closely related to GL *fuscans* than to GL 1.

Whole-genome sequencing is relatively cheap, easy and quick and readily discovers genetic variation that can be utilized as neutral molecular markers to track specific genotypes (Vinatzer et al., 2014; Goss, 2015). It can also reveal biologically interesting variation and the incidence and distribution of avirulence factors (e.g., T3SS effectors) across the pathogen population allowing for rational deployment of genetic resistance in host crop plants, as was recently proposed for cassava and its pathogen *X. axonopodis* pv. *manihotis* (Bart et al., 2012). Other authors have pointed out that deployment of resistance without an awareness of pathogenic variation within the pathogen population could result in costly failure (Taylor et al., 1996; Fourie and Herselman, 2011). At the time of writing (July 2015) sequence assemblies are publicly available for 379 *Xanthomonas* genomes. No genome sequences were currently available for *Xap*, but two *Xff* genome sequences have been published: a finished genome for strain 4834-R (Darrasse et al., 2013b) and a draft assembly for strain 4844 (Indiana et al., 2014). A previous review (Ryan et al., 2011) presented some of the insights into *Xanthomonas* biology revealed by genome sequencing.

In the current study, we aimed to exploit whole-genome sequencing to catalog genetic diversity of CBB pathogens within each of the MLSA-based genetic lineages from common bean and strains from lablab and Lima beans. We also hypothesized that there might be some genetic features that are shared between phylogenetically distant lineages of CBB pathogens that reflect genetic exchange or adaptation to a common host. Therefore, we sequenced and bioinformatically analyzed the genomes of 26 strains deposited in the strain collections as *Xap* or *Xff* spanning six continents and more than four decades.

The specific objectives of this study were:

- To determine the phylogenomic relationships between *Xap* and *Xff* bacterial strains from different host species: common, lablab and Lima beans.
- To identify genetic variation within *Xap* or within *Xff*. These sequence variations could be exploited as molecular markers for use in epidemiological and phylogeographic studies.
- To determine patterns of conservation and variation in the complement of T3SS effectors between and within *Xap* and *Xff*. This knowledge can inform future rational deployment of disease resistance genes in beans.
- To identify genes or alleles that have been recently transmitted between phylogenetically divergent CBB bacteria.
- Identify candidate genetic determinants of the fuscous genotype, i.e., production of the brown pigment on tyrosine-containing medium.

## Materials and methods

### Genome sequencing

Genomic DNA was prepared from overnight liquid cultures of bacteria revived from the NCPPB grown on Yeast extract-Dextrose-Calcium Carbonate solid medium (i.e., agar plates) for 2 days at 28°C. DNA extraction was performed using the QIAamp DNA Mini kit (Qiagen, Hilden, Germany) applying proteinase K incubation for 30 min. We used the Nextera XT kit (Illumina, San Diego, USA) for library preparation following manufacturer's instructions. Purification was carried after tagmentation using AMPure XP beads (Beckman Coulter, High Wycombe, UK) prior to pooling. The 15pM library was then sequenced on an Illumina MiSeq using reagent kit chemistry v3 with 600 cycles.

### Bioinformatics

#### Quality control on genomic sequence data

The quality of sequence data was checked using FastQC (Andrews)^1^ Poor-quality and adaptor-containing reads were filtered and trimmed using FastQ-MCF (Aronesty, 2011).

#### Alignment of sequence reads vs. a reference genome sequence

For alignment of genomic sequence reads against reference genome sequences of *Xff* 4834-R (Darrasse et al., 2013b) and *X. axonopodis* pv. *citri* 306 (Da Silva et al., 2002), we used BWA-MEM (Li, 2014). Resulting alignments were visualized using IGV (Thorvaldsdóttir et al., 2013).

#### Phylogenetic analysis and calling single-nucleotide variations

Phylogenetic analysis of the multi-locus sequence data was conducted in MEGA6 (Tamura et al., 2013). Multiple sequence alignments were performed using Muscle (Edgar, 2004). Evolutionary history was inferred using the maximum likelihood method based on the general time reversible model (Nei and Kumar, 2000). Initial tree(s) for the heuristic search were obtained by applying the Neighbor-Joining method to a matrix of pairwise distances estimated using the maximum composite likelihood (MCL) approach.

For phylogenetic analysis of whole-genome assemblies, we used the Parsnp program from the Harvest suite (Treangen et al., 2014). Phylogenetic trees generated from Parsnp in Newick format were imported into MEGA6 for preparation of the final figures. Parsnp uses FastTree2 to generate approximately maximum likelihood trees (Price et al., 2010). Distributions of single-nucleotide variations, calculated by Parsnp, were visualized using Gingr from the Harvest suite.

To check the reliability of the SNPs called by Parsnp, we further checked them using our previously described method (Mazzaglia et al., 2012; Wasukira et al., 2012; Clarke et al., 2015). For this method, we aligned the sequence reads against the reference genome sequence using BWA-mem version 0.7.5a-r405 (Li, 2013, 2014) with default parameter values and excluding any reads that did not map uniquely to a single site on the reference genome. From the resulting alignments, we generated pileup files using SAMtools version 0.1.19-96b5f2294a (Li et al., 2009). We then parsed the pileup-formatted alignments to examine the polymorphism status of each single-nucleotide site in the entire *Xff* 4834-R reference genome. For each single-nucleotide site we categorized it as either ambiguous or unambiguous. A site was considered to be un- ambiguous only if there was at least 5 × coverage by genomic sequence reads from each and every bacterial strain and only if for each and every bacterial strain, at least 95% of the aligned reads were in agreement. Any sites that did not satisfy these criteria were considered to be ambiguous and excluded from further analysis. Over the remaining unambiguous sites, we could be very confident in the genotype for all the sequenced strains.

#### De novo assembly

Prior to assembly, we combined overlapping reads using FLASH (Magoč and Salzberg, 2011). Genomes were assembled using SPAdes version 3.5.0 (Bankevich et al., 2012) with read error correction and with the “- - careful” switch. We assessed the quality of the assemblies and generated summary statistics using Quast (Gurevich et al., 2013) and REAPR (Hunt et al., 2013).

#### Automated annotation of genome assemblies

Genome assemblies were annotated via the Prokaryotic Genomes Automatic Annotation Pipeline (PGAAP) at the NCBI.

#### Comparison of gene content

To determine the presence or absence of genes in the newly sequenced genomes, we used alignment of genomic sequence reads against a reference pan-genome rather than comparison between genome assemblies. The reference pan-genome consisted of a set of gene sequences, each being a sole representative of a cluster of orthologous genes from all *Xanthomonas* genomes whose sequences were currently available; clustering of orthologous gene sequences was performed using UCLUST (Edgar, 2010). The reason for taking this approach (i.e., alignment of raw reads rather than alignment of assemblies) was to avoid potential errors arising from gaps in the genome assemblies. We aligned sequence reads against the reference genome sequence using BWA-MEM (Li and Durbin, 2009; Li, 2014) and used coverageBed from the BEDtools package (Quinlan and Hall, 2010) to determine the breadth of coverage of each gene in the resulting alignment. These breadths of coverage were visualized as heatmaps using the pheatmap module in R (R Development Core Team, 2013). We also compared genome assemblies using BRIG (Alikhan et al., 2011).

## Results

### Overview of sequencing results

We performed genomic re-sequencing on a collection of 26 *Xap* and *Xff* strains from the strain collections at NCPPB and CIAT as summarized in Table 1. For most of the strains, we obtained a depth of coverage of at least 40 x and thus were able to generate *de novo* genome assemblies. However, for seven of the genomes, there was less than 40 x coverage. We investigated the relationship between coverage depth and assembly quality by assembling subsets of the sequence reads from NCPPB 1058. We found that contig N_50_ length peaked at around 40 x coverage, with further increases in depth yielding little or no increase in contig lengths.

**Table 1:** **Sequenced strains**.

**Strain**	**Country and date of isolation**	**Host**	**Depth of coverage**	**Clade**	**Accession numbers**
*Xap* NCPPB 556 (LMG 829)	Sudan (Shambat) 1957	Lablab bean	15 x	“Lablab”	JTJF00000000 SRX1048889
*Xap* NCPPB 557 (LMG 830)	Sudan (Wad-Medani) 1957	Lablab bean	57 x	“Lablab”	JWTE00000000 SRX1048890
*Xap* NCPPB 2064 (LMG 8015)	Sudan (Wad-Medani) 1965	Lablab bean	108 x	“Lablab”	JSEZ00000000 SRX1048891
*Xap* NCPPB 1713 (LMG 8013,)	Zimbabwe 1962	Lablab bean	30 x	“Lablab”	JWTD00000000 SRX1048892
“*Xap*” NCPPB 3660	Brazil 1975	Common bean	63 x	“Fuscans”	JSEX00000000 SRX1050058
*Xff* NCPPB 381^*^ (LMG 826, CFBP 6165)	Canada 1957	Common bean	40 x	“Fuscans”	JTKK00000000 SRX1050059
*Xff* CIAT X621	South Africa (Cedan) 1995	Common bean	68 x	“Fuscans”	JXHS00000000 SRX1050082
*Xff* CIAT XCP631	Colombia 2004	Unknown	33 x	“Fuscans”	JXLW00000000 SRX1050252
*Xff* NCPPB 1056^*^ (LMG 7457)	Ethiopia 1961	Common bean	44 x	“Fuscans”	JSEV00000000 SRX1049856
“*Xap*” NCPPB 1058^**^	Ethiopia 1961	Common bean	150 x	“Fuscans”	JSEY00000000 SRX1049857
*Xff* NCPPB 1433^*^ (LMG 8016)	Hungary 1956	Common bean	51 x	“Fuscans”	JSBT00000000 SRX1049858
*Xff* NCPPB 2665^*^ (LMG 841)	Italy 1973	Common bean	70 x	“Fuscans”	JSBQ00000000 SRX1049859
*Xff* NCPPB 1654^*^ (LMG 837)	South Africa 1963	Common bean	93 x	“Fuscans”	JSBR00000000 SRX1049860
“*Xap”* NCPPB 670^**^ (LMG 832)	Uganda 1958	Common bean	32 x	“Fuscans”	JRRE00000000 SRX1049872
*Xff* NCPPB 1402^*^ (LMG 7459)	Uganda 1962	Common bean	17 x	“Fuscans”	JSEW00000000 SRX1049873
*Xff* NCPPB 1158^*^ (LMG 7458)	UK 1961	Common bean	40 x	“Fuscans”	JSBS00000000 SRX1049874
*Xff* NCPPB 1495^*^ (LMG 8017)	UK 1963	Common bean	30 x	“Fuscans”	JSEU00000000 SRX1049875
*Xap* NCPPB1646 (LMG 8011)	Australia 1964	Common bean	48 x	GL1	JTCT00000000 SRX1050299
*Xap* NCPPB 301	Canada pre-1951	Not known	50 x	GL1	JTCU00000000 SRX1050300
*Xap* CIAT XCP123	Colombia 1974	Lima bean	30 x	GL1	JXLV00000000 SRX1050292
*Xap* NCPPB 1420 (LMG 836)	Hungary 1956	Common bean	92 x	GL1	JTCV00000000 SRX1050301
*Xap* NCPPB 1811 (LMG 8014, CFBP 6164)	Romania 1966	Common bean	54 x	GL1	JWTF00000000 SRX1050302
*Xap* NCPPB 1680 (LMG 8012)	Tanzania 1964	Common bean	66 x	GL1	JWTG00000000 SRX1050303
*Xap* NCPPB 3035 (T) (LMG 7455, CFBP 6546)	USA pre-1978	Common bean	27 x	GL1	JSFA00000000 SRX1050305
*Xap* NCPPB 220 (NCTC 4331)	USA pre-1948	Not known	62 x	GL1	JWTH00000000 SRX1050306
*Xap* NCPPB 1138 (LMG 834)	Zambia 1961	Common bean	45 x	GL1	JWTI00000000 SRX1050323
“*Xap*” NCPPB 1128	Jamaica 1961	Common bean	80 x	Unknown *Xanthomonas* species	LFME00000000.1 SRX1090401

All strains had been deposited as Xap, except for those marked with an asterisk (

* ), which had been deposited as “X. axonopodis pv. phaseoli variant fuscans.” Strains marked with two asterisks (

** ) were deposited as Xap but are reported to produce brown pigment, according to the accession cards that were submitted along with the strains into the NCPPB. Depth of coverage was estimated from alignments of raw sequence reads against the reference genome of X. axonopodis pv. citri 306 (Da Silva et al., 2002) using BWA-MEM (Li and Durbin, 2009; Li, 2014). GenBank accession numbers are given for the genome assemblies and SRA accession numbers are given for the raw sequence reads. Accession numbers are given for synonymous strains from the Belgian Coordinated Collections of Micro-Organisms (LMG), the Collection Française de Bactéries associées aux Plantes (CFBP) and National Collection of Type Cultures (NCTC).

**Figure 1:**
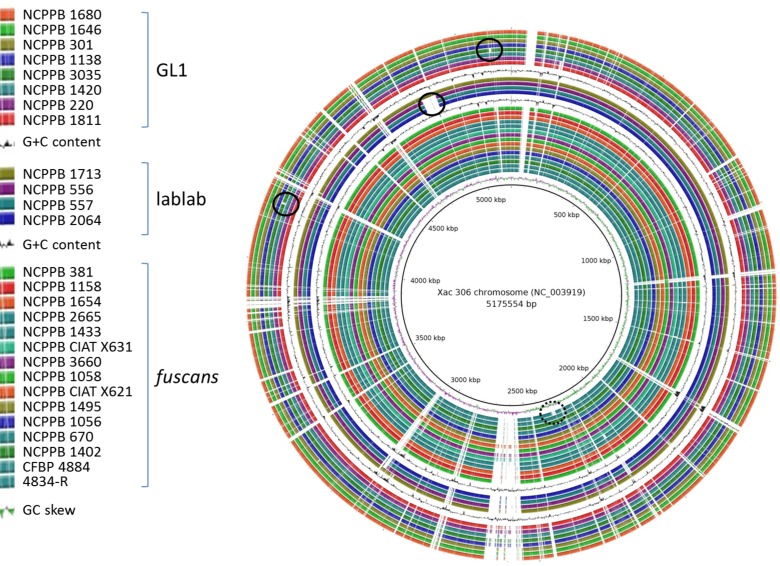
**Overview of genomic conservation among ***Xap*** and ***Xff*****. The newly sequenced genome sequences and those of previously sequenced Xff strains 4834-R (Darrasse et al., 2013b) and CFBP 4884 (Indiana et al., 2014) were aligned against the *Xac* 306 chromosome reference sequence (Da Silva et al., 2002) using BLASTN with an E-value threshold of 1 × 10^−6^. The alignments are visualized using BLAST Ring Image Generator (BRIG) (Alikhan et al., 2011). The innermost ring indicates the position on the reference chromosome. Positions covered by BLASTN alignments are indicated with a solid color; whitespace gaps represent genomic regions not covered by the BLASTN alignments. For clarity, the three genetic lineages (GL 1, lablab-associated strains and GL *fuscans*) are separated by repetitions of the plot of G+C content. The black circle indicates the previously reported (Darrasse et al., 2013b) absence of the flagellar gene cluster in *Xff* 4834-R. The solid-lined black circles indicate strain-specific genomic deletions observed in the present study.

Figure 1 shows an overview of the *de novo* assemblies of each sequenced *Xap* and *Xff* genome aligned against that of the *X. axonopodis* pv. *citri* 306 (Da Silva et al., 2002). See also the Supplementary Figures for genome-wide alignments of the assemblies using Mauve (Darling et al., 2004). Note that the 26 *Xap* and *Xff* genomes were assembled *de novo*, using SPAdes (Bankevich et al., 2012), without use of a reference sequence.

The contiguities of the assemblies were comparable to those of previously sequenced *Xanthomonas* genomes. This is illustrated by the distribution of N_50_ contig lengths, which ranged from 39.4 to 123.6 kb. The range for a recent study of 65 *X. axonopodis* pv. *manihotis* was 7.4–111.0 kb (Bart et al., 2012). A full summary of assembly statistics, calculated using Quast (Gurevich et al., 2013), is provided in the Supplementary Table S1.

Contiguity of an assembly does not necessarily correlate with accuracy. Therefore, in addition to the Quast analysis of assembly contiguity, we also assessed the accuracies of the assemblies using REAPR (Hunt et al., 2013). This method is based on aligning to the assembly the sequence reads from which it was generated. This allows detection of anomalies in coverage of the assembly by reads and flags two classes of potential errors: fragment coverage distribution (FCD) errors and low fragment coverage errors. We compared the frequencies of these two classes of potential error for each of our genome assemblies and also for each of the 65 previously published *X. axonopodis* pv. *manihotis* assemblies (Bart et al., 2012); see Supplementary Figure S1. The genome assemblies generated in the present study were of comparable quality to those from the previously published study. However, there is a general trend toward our genome assemblies having more “low fragment coverage” errors and fewer “FCD” errors.

**Figure 2:**
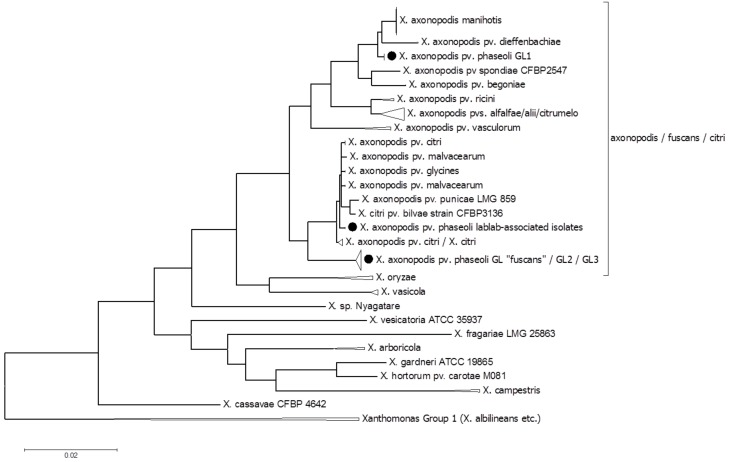
**Multi-locus sequence analysis to determine the phylogenetic positions of the sequenced strains within the species ***X. axonopodis*** and ***X. fuscans*****. The phylogenetic tree is based on alignment of six concatenated gene sequences (*atpD, dnaK, efp, fyuA, glnA*, and *gyrB*). The sequences for NCPPB 220, 301, 1138, 1420, 1646, 1680, 1811, 3035, and CIAT XCP123 were identical to those of CFBP 412, 6164, 6546, 6982, 6983, 6984, and 6985, which are classified as belonging to “pv. *phaseoli* GL1” (Alavi et al., 2008; Mhedbi-Hajri et al., 2013) and genetic group 9.4 (Rademaker et al., 2005). The sequences for NCPPB 381, 670, 1056, 1058, 1158, 1402, 1433, 1495, 1654, 2665, 3660, and CIAT X621 were identical to those of CFBP 1845, and 4834-R, which are classified as “pv. *phaseoli* GL *fuscans”* (Alavi et al., 2008; Mhedbi-Hajri et al., 2013) and genetic group 9.6 (Rademaker et al., 2005). The evolutionary history was inferred by using the Maximum Likelihood method based on the General Time Reversible model (Nei and Kumar, 2000). The tree with the highest log likelihood (−17100.2449) is shown. The percentage of trees in which the associated taxa clustered together is shown next to the branches. Initial tree (s) for the heuristic search were obtained by applying the Neighbor-Joining method to a matrix of pairwise distances estimated using the Maximum Composite Likelihood (MCL) approach. The tree is drawn to scale, with branch lengths measured in the number of substitutions per site. The analysis involved 284 nucleotide sequences. All positions containing gaps and missing data were eliminated. There were a total of 2697 positions in the final dataset. Evolutionary analyses were conducted in MEGA6 (Tamura et al., 2013).

To ascertain the phylogenetic positions of each sequenced strain, we initially used a multi-locus sequence analysis (MLSA) approach, using concatenated sequences from six genes that had been used in previous MLSA studies (Young et al., 2008; Almeida et al., 2010; Hajri et al., 2012; Hamza et al., 2012). This approach had the advantage that we could include in the analysis many *Xap* strains and other xanthomonads whose genomes had not been sequenced but for which MLSA data were available. Nucleotide sequences are available for these six genes from a large number of xanthomonads, either from whole-genome sequence assemblies or from the MLSA studies. We combined the publicly available sequences with homologous sequences extracted from the genomes newly sequenced for this study. The results of the MLSA revealed that the newly sequenced *Xap* and *Xff* genomes each fell into one of three distinct clades: GL 1, GL *fuscans* and a previously undescribed lineage associated with lablab bean (Figure 2).

The newly sequenced strains from lablab bean comprised a third clade, quite distinct from both *Xap* GL1 and from GL *fuscans* and indeed all previously described lineages of bean pathogens. The lablab-associated strains are closely related to members of Rademaker's genetic group 9.5, along with strains of pathovars *bilvae, citri, malvacearum*, and *mangiferaeindicae* that are pathogens of diverse plants including Bengal quince, *Citrus* spp., cotton and mango respectively (Bradbury, 1986; Rademaker et al., 2005). Also falling within this MLSA-based clade are strains of *X. axonopodis* pv. *glycines*, causative agent of bacterial pustule in soybean (Jones, 1987).

### Genome-wide SNP analysis elucidates phylogeny at greater resolution

**Figure 3:**
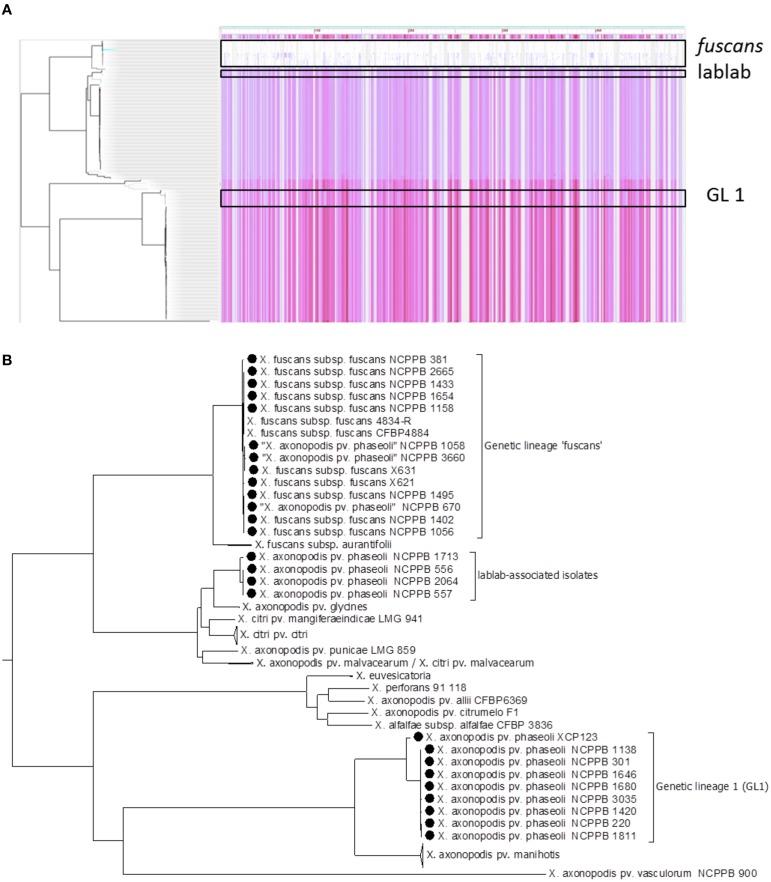
**Genome-wide identification of single-nucleotide variations**. Panel **(A)** shows a density plot of single-nucleotide variations paired with a phylogenetic tree, both generated using Harvest (Treangen et al., 2014) with the chromosome of Xff 4834-R (Darrasse et al., 2013b) as the reference sequence. Panel **(B)** shows the same phylogenetic tree as in Panel **(A)** but with taxon labels and some clades collapsed for clarity. In addition to the 26 newly sequenced genomes, all 134 publicly available genome assemblies from *X. axonopodis, X. fuscans, X. citri*, and *X. euvesicatoria* were included.

Based on six-gene MLSA alone, strains could be ascribed to one of the three genetic lineages (GL 1, GL *fuscans*, and GL lablab). However, genome-wide sequence comparisons provided additional resolution and revealed distinct clades (or sub-lineages) within each lineage. Figure 3A illustrates the distribution of single-nucleotide variations across the reference sequence of the chromosome of *Xff* 4834-R for 160 publicly available related genome sequences. Figure 3B shows a phylogenetic reconstruction of those 160 genomes based on those variants. Consistent with the MLSA results, the strains sequenced in the present study similarly fell into three clades.

**Figure 4:**
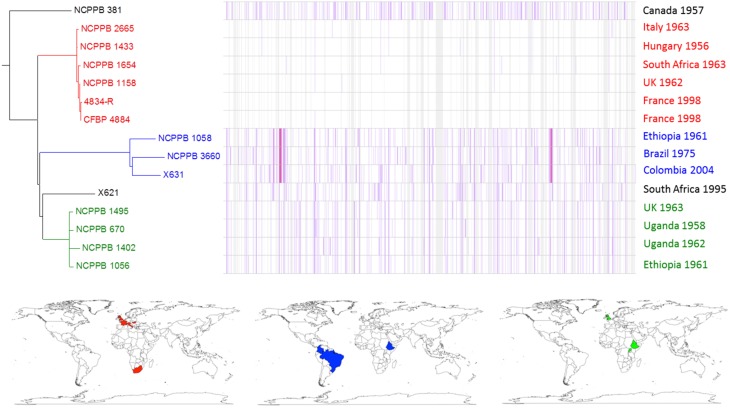
**Single-nucleotide variation within ***fuscans*** genetic lineage**. A density plot of single-nucleotide variations is shown paired with a phylogenetic tree, both generated using Harvest (Treangen et al., 2014) with the chromosome of *Xff* 4834-R (Darrasse et al., 2013b) as the reference sequence. The country and year of isolation is indicated for each bacterial strain. Three multi-strain sub-lineages are indicated by coloring in red, blue, and green respectively. The geographical locations of the countries of isolation are indicated on the world maps for each of the three sub-lineages, again colored respectively in red, blue, or green.

Within the *fuscans* lineage, the genome-wide comparison revealed at least three distinct sub-lineages, depicted in Figure 4 in red, blue and green respectively. Each of these three lineages includes strains from diverse geographical locations and years. For example, one sub-lineage includes strains from France (1998), Hungary (1956), Italy (1963), South Africa (1963) and the UK (1962). This suggests that this sub-lineage has been circulating in Europe for nearly six decades and has spread between Europe and South Africa at least once, perhaps indirectly via another locality. This pattern is consistent with spread of the pathogen via global trade of seeds.

The genome-wide sequence analysis also reveals that multiple genetic lineages may be present within a single geographical area. For example, NCPPB 1056 and NCPPB 1058 were both isolated in the same country and the same year (Ethiopia, 1961) and fall into two distinct sub-lineages (Figure 4).

**Figure 5:**
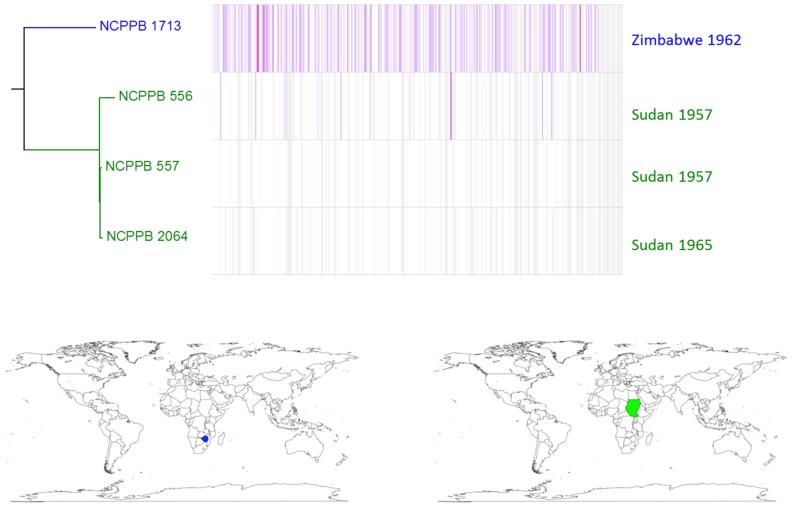
**Single-nucleotide variation among lablab-associated strains**. A density plot of single-nucleotide variations is shown paired with a phylogenetic tree, both generated using Harvest (Treangen et al., 2014) with the chromosome of NCPPB 557 as the reference sequence. The country and year of isolation is indicated for each bacterial strain. Two sub-lineages are indicated by coloring in blue (a single strain) and red (three strains) respectively. The geographical locations of the countries of isolation are indicated on the world maps for each of the three sub-lineages, again colored respectively in blue or red.

**Figure 6:**
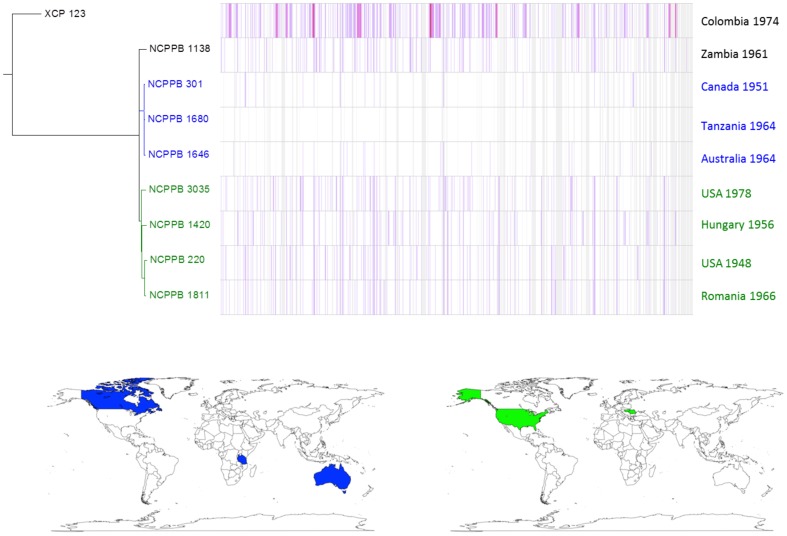
**Single-nucleotide variation within ***Xap*** GL 1**. A density plot of single-nucleotide variations is shown paired with a phylogenetic tree, both generated using Harvest (Treangen et al., 2014) with the chromosome of NCPPB 1680 as the reference sequence. The country and year of isolation is indicated for each bacterial strain. Two multi-strain sub-lineages are indicated by coloring in blue and red respectively. The geographical locations of the countries of isolation are indicated on the world maps for each of the three sub-lineages, again colored respectively in blue or red.

Similar, intra-lineage variation can be observed for strains within the lablab-associated strains (Figure 5) and GL 1 (Figure 6). Among lablab-associated strains, those collected in Sudan between 1957 and 1965 cluster together and are distinct from NCPPB 1713, which originates from Zimbabwe in 1962. Within GL 1, there are two multi-strain sub-lineages, which are indicated in blue and green in Figure 5. The former sub-lineage spans Australia, Canada, and Tanzania. The latter sub-lineage includes strains from Hungary, Romania, and the USA. Strain NCPPB 1138 (from Zambia, 1961) is distinct from both of these. The single GL 1 strain from Lima bean (CIAT XCP123, Colombia, 1974) is distinct from all of the strains from common bean (Figure 5); however, based on MLSA alone, it is indistinguishable from the other GL 1 strains.

Across the 4,981,995-bp chromosome sequence of *Xff* 4834-R, Harvest identified a total of 135,321 SNPs. This number includes all single-nucleotide sites that show variation between any of the 160 genome assemblies included in the analysis. A subset of 61,462 of those SNPs showed polymorphism among the 26 *Xap* and *Xff* genomes sequenced in the present study. The Harvest SNP calling takes as its input assembled genome sequences. Thus, substitution errors in the assemblies then could appear as false positives. Gaps in the assemblies are unlikely to generate false-positive SNP calls as Harvest only considers the core genome, i.e., those regions of the genome that are present in all of the genome assemblies and discards genomic regions present in only a subset of the assemblies. To assess the reliability of the Harvest SNP calls, we compared the results with a read-based method of SNP calling that we have used previously (Mazzaglia et al., 2012; Wasukira et al., 2012; Clarke et al., 2015). Read-based methods have the advantage of not being reliant on assembly and they exploit the signal from multiple independent overlapping sequence reads at each site in the genome sequence. However, sequence reads are not available for the majority of *Xanthomonas* genome sequences, since for most studies only the assemblies and not the reads have been deposited in the public repositories. Of the 61,462 SNPs that Harvest called for the *Xap* and *Xff* genomes, our read-based method confirmed 53,811 (87.5%).

It is evident from Figures 3–6 that single-nucleotide variations occur throughout the chromosome. However, the distribution is not uniform and there are several apparent “hotspots” of variation. The most likely explanation for these regions of higher-than-average sequence divergence is horizontal acquisition of genetic material from relatively distantly related strains. Such incongruent patterns of sequence similarity due to horizontal transfer have been reported previously in *Xanthomonas* species (Fargier et al., 2011; Hamza et al., 2012).

### Gene-content varies between and within each clade

**Figure 7:**
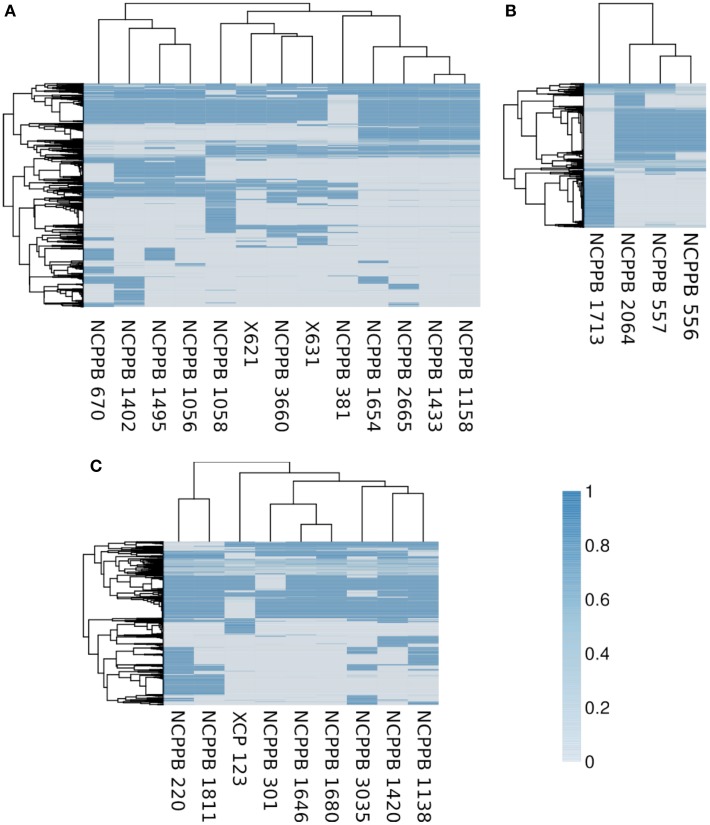
**Variation in gene content within each genetic lineage**. The heatmaps essentially indicate the presence or absence of each gene in each sequenced genome. To determine the breadth of coverage of a gene, the genomic raw sequence reads are aligned against a reference pan-genome using BWA-MEM (Li, 2013, 2014) and the breadth of coverage is calculated using coverageBed (Quinlan and Hall, 2010). A coverage of one indicates complete coverage of the gene by aligned genomic sequence reads, indicating presence of the gene. A coverage of zero indicates that no genomic sequence reads matched the gene, indicating that it is absent (or at least highly divergent in sequence). In each heatmap, the genomes (columns) are clustered according to gene content and the genes (rows) are clustered according to their patterns of presence and absence across the genomes. The core genome, i.e., the subset of genes that are present in all strains, is excluded from the heatmap. **(A)** Summarizes the pattern of presence and absence for 1188 genes in the *fuscans* lineage, **(B)** for 472 genes in lablab-associated strains and **(C)** for 535 genes in *Xap* GL 1. Note that each gene is the representative of a cluster of orthologous genes.

Consistent with the indications of horizontal genetic transfer described in the previous section, we observed significant variations in gene presence and absence among strains within each of the three genetic lineages (Figure 7). Within the *fuscans* strains, there were 1188 clusters of orthologous genes that were present in at least one strain and absent from at least one other (Figure 7A). Among the lablab-associated strains, 472 orthologous gene clusters showed presence-absence polymorphism (Figure 7B). Among GL 1, the number was 535 (Figure 7A). Clustering of genomes according to gene content is broadly congruent with phylogeny. Supplementary Tables S2–S7 list genes whose presence distinguishes between Xff, Xap GL 1 and lablab-associated strains. Additionally, the four lablab-associated strains all contain six genes that have no close homologs amongst other sequenced xanthomonads. These are predicted to encode: three hypothetical proteins (KHS05433.1, KKY05378.1, and KHS05434.1), pilus assembly protein PilW (KHS05489.1), an oxidoreductase (KHS05432.1), and an epimerase (KHS05485.1).

### Strain-specific large chromosomal deletions

A large chromosomal deletion has been previously reported in *Xff* 4834-R in which a large part of the flagellar gene cluster is absent (Darrasse et al., 2013b). This deletion is visible in Figure 1 at around position 2310 kb in the *Xac* 306 chromosome and indicated by a black circle with broken line. Although, similar deletions were reported in 5% of the strains tested (Darrasse et al., 2013b), this flagellar gene cluster was intact in all of the genomes sequenced in the current study as well as in the previously sequenced *Xff* 4884 (Indiana et al., 2014).

In addition to the strain-specific flagellar deletion, Figure 1 reveals several other large genomic deletions, examples of which are indicated with black circles. The largest example is a 50-kb region of the *Xac* 306 chromosome sequence that is absent from the three Sudanese lablab-associated strains but present in the Zimbabwe strain. This absence is visible in Figure 1 at between 4.82 and 4.87 Mb on the reference chromosome sequence and indicated by a black circle. The absence of this region is supported not only by the *de novo* assemblies of NCPPB 556, 557 and 2064, but also by alignment of the raw sequence reads against the *Xac* 306 reference genome, eliminating the possibility that it merely represents an assembly artifact. This region is illustrated in Supplementary Figure S6, includes locus tags XAC4111–XAC4147 and is predicted to encode a type-6 secretion system (Darrasse et al., 2013b).

Other examples include a deletion of approximately 9 Kb that is deleted in *Xap* NCPPB 3035, resulting in loss of its ortholog of gene XAC RS17930 and parts of the two flanking genes XAC_RS17925 and XAC_RS17935 at around position 4.20 Mb on the reference genome (Supplementary Figure S7). A second example of a deletion unique to NCPPB 3035 spans approximately 10 kb at around position 5.10 Mb (Supplementary Figure S8). The deleted region contains genes XAC_RS21755 (predicted plasmid stabilization protein) to XAC_RS21815 (predicted transposase) and likely represents a mobile element.

### Strains of *Xap* GL1 encode a SPI-1-like T3SS

**Figure 8:**
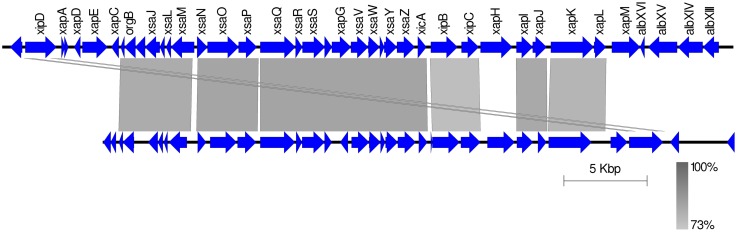
**The SPI-1-like T3SS in ***Xap*** GL 1**. The figure shows a TBLASTN alignment of the *X. albilineans* chromosome (Pieretti et al., 2009) vs. the genome of *Xap* NCPPB 3035 (sequenced in the present study). Sequence identity is indicated by the black-gray-white color scale.

A previously published suppression subtractive hybridizations study comparing bean pathogens and closely related xanthomonads revealed the presence of genes encoding several protein components of a T3SS similar to that of *Salmonella* pathogenicity island 1 (SPI-1) in the genome of *Xap* CFBP 6164 (Alavi et al., 2008). This strain is synonymous with NCPPB 1811 and belongs to lineage GL 1. Subsequently, genome sequencing revealed that *X. albilineans* encodes a SPI-1-like T3SS (Pieretti et al., 2009, 2015) and targeted sequencing confirmed its presence in two further *Xap* GL 1 strains: CFBP 2534 (same as NCPPB 3035) and CFBP 6982 (Marguerettaz et al., 2011). Whole-genome sequencing in the current study indicated that this SPI-1-like T3SS was encoded in the genomes of all GL 1 strains from common bean and Lima bean (Figure 8) but was absent from GL fuscans and from the lablab-associated strains. All of the putative structural genes for the T3SS are conserved in *Xap* GL 1 but the *xapABCDEFGH* genes, hypothesized to encode effectors that are substrates of the T3SS in *X. albilineans* (Marguerettaz et al., 2011), are not conserved in *Xap*.

### Repertoires of Hrp T3SS effectors

**Figure 9:**
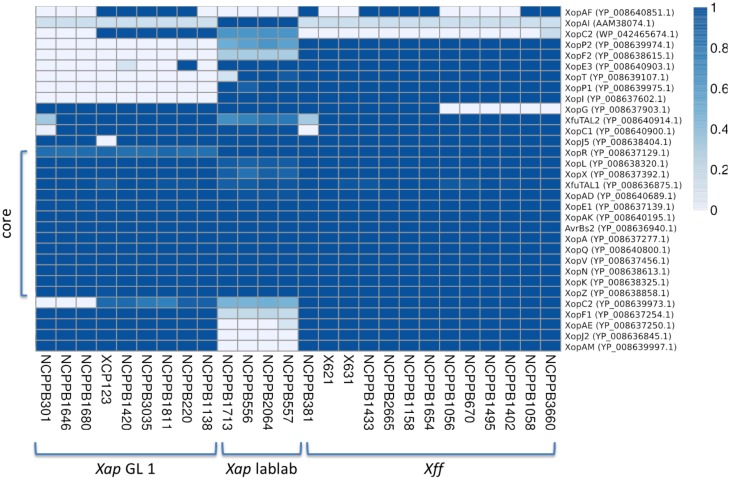
**Repertoires of T3SS effectors**. The heatmap indicates the proportion covered by aligned genomic sequence reads over each T3SS effector gene DNA sequence. GenBank accession numbers are given on the right side. Genomic sequence reads were aligned against the effector gene sequences using BWA-MEM. A coverage of 1.0 represents complete coverage, indicating that the gene is present in the respective genome. A coverage of 0.0 represents a complete absence of aligned sequence reads, indicating absence of the gene from the respective genome.

Previous genome sequencing of *Xff* 4834-R revealed the presence of genes encoding 30 predicted effectors potentially secreted by the Hrp T3SS (Darrasse et al., 2013b). We searched for orthologs of these and other *Xanthomonas* T3SS effectors in the newly sequenced *Xap* and *Xff* genomes using TBLASTN (Altschul et al., 1990) to search the genome assemblies against each protein query sequence. The results are summarized in Figure 9. There is a core set of 14 effectors that is encoded in all sequenced strains of *Xap* and *Xff* : XopK, XopZ, XopR, XopV, XopE1, XopN, XopQ, XopAK, XopA, XopL, AvrBs2, and XopX. Four of these are also included in the core set of effectors conserved among 65 strains of *X. axonopodis* pv. *manihotis* (Bart et al., 2012), namely XopE1, XopL, XopN, and XopV. Several others are encoded in most but not all of the newly sequenced genomes, for example: XopC1, XfuTAL2, and XopJ5. Others appear to be limited to just one of the three lineages. For example, XopF2 is limited to lineage *fuscans*, XopC2 is found only in *Xap* GL1 and XopAI is restricted to the lablab-associated strains.

### The molecular basis for pigmentation

**Figure 10:**
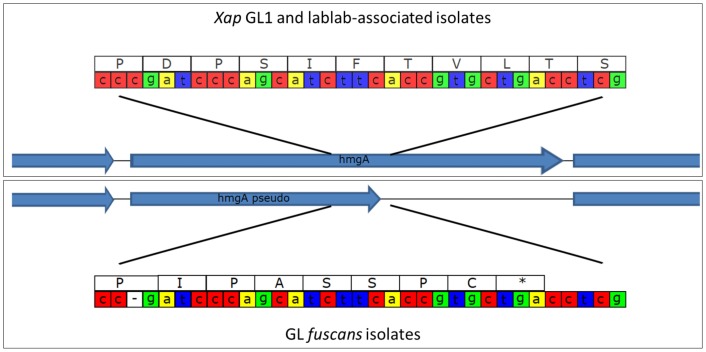
**Disruption of the ***hmgA*** gene in GL ***fuscans*****. The cartoon illustrates a sequence polymorphism in the hmgA gene whereby all of the sequenced GL *fuscans* strains (including previously sequenced 4834-R and CFBP4884) have a single-nucleotide deletion that results in a frame-shift and premature stop codon. All of the *Xap* GL 1 and lablab-associated strains encode a full-length protein product.

Some bacterial strains from CBB infections produce a brown pigment when grown in tyrosine-containing medium and are therefore described as “fuscous.” The pigment is not believed to be directly associated with virulence (Gilbertson et al., 1991; Fourie, 2002) but fuscous strains tend to be very virulent on bean (Birch et al., 1997; Toth et al., 1998). The brown color arises from oxidized homogentisic acid (2,5 dihydroxyphenyl acetic acid), an intermediate in the tyrosine catabolic pathway that gets secreted and oxidized in these fuscous strains (Goodwin and Sopher, 1994). Genome sequencing of the fuscous strain *Xff* 4834-R revealed a single-nucleotide deletion in *hmgA*, the gene encoding homogentisate oxygenase (Darrasse et al., 2013b). This enzyme catalyzes a step in the tyrosine degradation pathway that converts tyrosine to fumarate and hence its inactivation likely disrupts tyrosine degradation leading to accumulation of homogentisate and its subsequent oxidation to form the brown pigment. Consistent with this hypothesis, we found that the single-nucleotide deletion was present in all of the sequenced strains belonging to GL *fuscans* resulting in a predicted protein product that is truncated, while the *hmgA* gene was intact in all of the *Xap* GL1 and lablab-associated *Xap* genomes (see Figure 10).

### Recent genetic exchange between *Xap* GL 1 and GL fuscans

Patterns of single-nucleotide variation (Figure 3A) revealed some regions of the genome where *Xap* GL 1 had many fewer variants with respect to the *Xff* 4384-R reference genome than did the closely related *X. axonopodis* pv. *manihotis*. Closer inspection revealed numerous genes where the *Xap* GL 1 strains shared an identical allele with *Xff*, a pattern that is incongruent with their relatively distant phylogenetic relationship.

**Figure 11:**
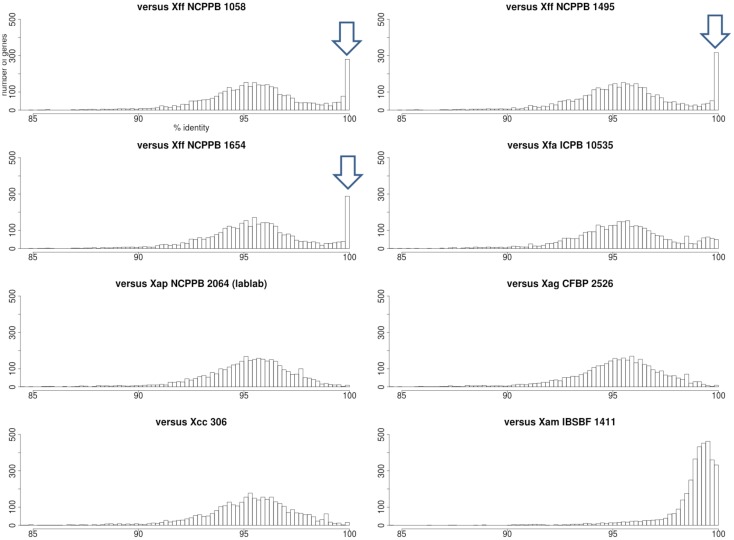
**Nucleotide sequence identities of ***Xap*** GL 1 genes vs. other lineages and pathovars**. Each histogram shows the frequency distribution of sequence identity between genes from a *Xap* GL 1 strain (NCPPB 1680) and their closest BLASTN matches in other lineages including GL *fuscans* (NCPPB 1058, 1494 and 1654), lablab-associated *Xap* (NCPPB 2064), X. *fuscans* subsp. *aurantifolii* (ICPB 10535), *X. axonopodis* pv. *glycines* (CFBP 2526), *X. axonopodis* pv. *citri* (306) and *X. axonopodis* pv. *manihotis* (IBSBF 1411) (Da Silva et al., 2002; Moreira et al., 2010; Bart et al., 2012; Darrasse et al., 2013a). The arrowheads indicate the positions of the peaks at 100% sequence identity between GL 1 and GL *fuscans*.

To further investigate this phenomenon, we calculated pairwise nucleotide sequence identities for each *Xap* GL 1 gene vs. its closest homolog in other lineages within *X. axonopodis* and *X. fuscans*. The results are summarized in Figure 11. Pairwise sequence identities between *Xap* GL 1 and *Xff* (GL *fuscans*) followed a bimodal distribution with peaks at around 96% and at 100%. The peak at 100% was not observed for identities between *Xap* GL 1 and other lineages (*X. axonopodis* pv. *glycines, X. axonopodis* pv. *citri, X. axonopodis* pv. *manihotis, X. fuscans* subsp. *Aurantifolii*, and lablab-associated *Xap*). Table 2 lists examples of genes with 100% identity between *Xap* GL 1 and *Xff*. Essentially the same set of genes is affected in all of the *Xap* GL 1 strains and the alleles are more similar to alleles from pathovars *citri* and *glycines* than to *manihotis*. Therefore, the most parsimonious explanation is that these alleles have been acquired by the ancestors of *Xap* GL 1 from the *fuscans* lineage.

**Table 2:** **Genes in ***Xap*** GL 1 that share 100% nucleotide sequence identity with ***Xff*** (GL ***fuscans***)**.

**GenBank accession number and predicted product of *Xap* NCPPB 1680**	***Xag* CFBP 2526 (%)**	***Xam* IBSBF 1411 (%)**	***Xap* NCPPB2064 (%)**	***Xfa* ICPB 10535 (%)**
KHS20952 glutamine amidotransferase	97.73	99.47	97.61	98.14
KHS20955 preprotein translocase	97.28	94.42	98.88	99.20
KHS21044 phospholipase	96.36	93.73	96.49	99.75
KHS21241 ATPase AAA	97.92	93.84	97.92	98.67
KHS21242 histidine kinase	96.95	93.91	98.54	99.58
KHS21578 preprotein translocase subunit SecE	98.28	95.58	98.03	99.51
KHS21579 transcription antiterminator NusG	98.03	94.97	98.03	99.82
KHS21580 50S ribosomal protein L11	98.36	97.20	98.13	100.00
KHS21581 50S ribosomal protein L1	97.71	98.14	97.56	99.28
KHS21586 30S ribosomal protein S12	99.20	98.66	98.93	100.00
KHS22189 membrane protein	94.84	93.28	0.00	99.06
KHS22203 type VI secretion protein	97.93	96.69	0.00	99.17
KHS22241 UDP-2 3-diacylglucosamine hydrolase	98.25	95.42	98.12	99.46
KHS22843 cardiolipin synthetase	94.63	94.94	94.63	99.68
KHS23016 ribose-phosphate pyrophosphokinase	98.02	97.08	98.33	99.37
KHS23018 peptidyl-tRNA hydrolase	96.98	95.21	96.98	98.80
KHS23156 heat shock protein GrpE	99.23	95.56	99.23	99.81
KHS23251 membrane protein	98.83	97.96	98.98	99.56
KHS23252 membrane protein	97.67	94.25	97.67	99.46
KHS23254 methionine ABC transporter substrate-binding	97.78	95.64	97.78	98.75
KHS23255 metal ABC transporter permease	99.14	96.09	99.14	99.86
KHS23256 methionine ABC transporter ATP-binding protein	97.12	95.13	97.02	99.21
KHS23257 membrane protein	97.43	97.70	97.54	97.66
KHS23258 nucleotide-binding protein	98.56	96.91	98.14	99.38
KHS23269 type III secretion system effector protein XopAK	94.94	88.47	94.94	97.12
KHS23489 phosphomethylpyrimidine kinase	98.15	95.67	98.15	99.88
KHS23490 membrane protein	98.51	95.23	98.06	99.85
KHS23713 S-adenosylmethionine synthetase	99.01	98.02	98.92	99.83
KHS24197 molybdopterin-guanine dinucleotide biosynthesis	97.54	94.02	97.36	99.47
KHS24815 50S ribosomal protein L6	95.07	96.94	96.02	95.83
KHS24816 30S ribosomal protein S8	98.24	99.25	98.24	98.24
KHS24818 50S ribosomal protein L5	97.97	98.52	97.97	99.26
KHS24819 50S ribosomal protein L24	100.00	99.68	100.00	100.00
KHS24820 50S ribosomal protein L14	99.73	98.91	98.91	99.46
KHS24821 30S ribosomal protein S17	99.26	98.14	98.88	99.63
KHS24822 50S ribosomal protein L29	99.46	97.84	99.46	99.46
KHS25207 histone-like nucleoid-structuring protein	98.50	96.26	97.76	99.75
KHS25209 membrane protein	96.86	95.48	96.86	98.82
KHS25211 3-methyladenine DNA glycosylase	95.97	93.71	95.65	99.19
KHS25388 EF hand domain-containing protein	98.02	93.65	98.02	98.58
KHS25405 thioredoxin	99.12	97.36	98.83	100.00
KHS25407 ABC transporter	99.42	97.67	99.42	100.00
KHS25490 cupin	96.29	93.98	96.20	98.95
KHS25537 peptide ABC transporter permease	98.81	96.57	98.81	99.60
KHS26053 RNA-binding protein	98.35	96.69	99.17	99.59
KHS26541 3-demethylubiquinone-9 3-methyltransferase	97.47	98.61	97.47	97.61
KHS26908 lytic transglycosylase	95.53	86.24	94.99	99.11
KHS26912 membrane protein	97.87	96.17	97.77	99.36
KHS26916 malto-oligosyltrehalose trehalohydrolase	97.32	93.65	96.93	99.49
KHS27359 S-(hydroxymethyl)glutathione dehydrogenase	97.92	94.94	97.83	99.82
KHS27633 Crp/Fnr family transcriptional regulator	95.80	94.35	95.66	99.47
KHS27636 ATP phosphoribosyltransferase	96.28	95.30	96.50	99.67
KHS27638 histidinol-phosphate aminotransferase	96.70	90.87	97.07	99.45
KHS27639 imidazoleglycerol-phosphate dehydratase	95.21	93.17	95.12	99.65
KHS27640 imidazole glycerol phosphate synthase	95.18	93.52	95.02	99.67
KHS28695 GntR family transcriptional regulator	97.79	97.51	97.79	99.17
KHS28696 vitamin B12 ABC transporter permease	98.20	96.41	98.38	99.28
KHS28992 NlpC-P60 family protein	94.19	91.30	94.48	97.88
KHS28993 ErfK/YbiS/YcfS/YnhG family protein	96.65	91.75	96.13	99.48
KHS29058 membrane protein	98.59	95.07	97.54	100.00
KHS29941 CDP-diacylglycerol–serine	99.22	97.02	98.97	99.74
KKY054115 membrane protein	96.92	94.62	94.62	99.23
KKY05325 membrane protein	100.00	93.75	100.00	100.00

Each of the Xap NCPPB 1680 genes shares 100% identity over at least 95% of its length with its ortholog in Xff strains NCPPB 1058, 1495, and 1654. For comparison, percentage nucleotide sequence identities are given for each gene vs. X. axonopodis pv. manihotis (Xam), lablab-associated X. axonopodis pv. phaseoli (Xap).

Genome sequencing of *Xap* strains from lablab bean has revealed a previously unknown distinct lineage of *Xap*. This lineage is more closely related to strains of *X. axonopodis* pv. *glycines* that to any of the previously described genetic lineages of *Xap*. The existence of a separate lablab-associated lineage on lablab suggests that there may not be frequent movement of CBB bacteria between this species and common bean. However, conformation of this hypothesis will require genotyping of larger numbers of strains; with the availability of these genome data it will be straightforward to design PCR-based assays to identify bacterial strains belonging to this newly discovered lineage.

It was previously observed that a *Xap* strain from common bean (NCPPB 302) was less pathogenic on lablab than bacteria isolated from naturally infected lablab (Sabet, 1959). The same study also reported that the *Xap* strains (Dol1, 2 and 3) were less pathogenic on common bean than was *Xap* NCPPB 302, hinting at the presence of distinct populations of *Xap* differentially adapted to different host species. Furthermore, a subsequent study found that *Xap* strain Dol 3, isolated from lablab in Medani, Sudan, 1965, was pathogenic only on common bean and lablab bean; it was not pathogenic on any of the other leguminous plants that were tested, including several *Vigna* spp., *Rhynchosia memnonia*, mungo bean, pigeon pea, alfalfa, butterfly pea, velvet bean, pea, and white lupin.

To the best of our knowledge, no recent quantitative data are available for the extent and severity of common bacterial blight on lablab. However, in 1959, leaf blight on this crop was reported as widespread and often severe in the Gezira and central Sudan (Sabet, 1959).

The single sequenced bacterial strain from Lima bean clearly fell within *Xap* GL 1, along with strains from common bean, including the pathovar type strain (NCPPB 3035). However, genome-wide phylogenetic reconstruction revealed that the Lima-associated strain was the most early-branching within this lineage and suggests that it has been genetically isolated from the population that is geographically widely dispersed on common bean (Figure 6). Again, the availability of these genomic data will facilitate development of PCR-based assays to rapidly genotype larger panels of strains to elucidate the population genetics.

The newly sequenced genomes confirm and extend previous observations (Alavi et al., 2008; Marguerettaz et al., 2011; Egan et al., 2014), suggesting that a SPI-1-like T3SS is probably universal among *Xap* GL 1 but absent from *Xff* and from the newly discovered lablab-associated lineage. We also confirm that a frame-shift in the *hmgA* gene, resulting in a presumably defective homogentisate 1,2-dioxygenase, is common to all sequenced strains of *Xff* and probably explains the accumulation of brown pigment in fuscous strains (Darrasse et al., 2013b). The *hmgA* gene appeared to be intact in all the GL 1 and lablab-associated strains consistent with the absence of report of pigment in these.

Previous comparative genomics studies of *Xanthomonas* species have highlighted the presence of rearrangements of fragments of the genome (Qian et al., 2005; Darrasse et al., 2013a). We observed no evidence of such rearrangements among the *Xff*, *Xap* GL1 nor among the lablab-associated *Xap* genomes sequenced in the present study (See Supplementary Figures S3–S5). However, the lack of evidence should not be interpreted as meaning that there are no such rearrangements; draft-quality genome assemblies, such as those generated in the present other related studies (Bart et al., 2012; Indiana et al., 2014; Schwartz et al., 2015), are fragmented into multiple contigs and/or scaffolds and if the breakpoints in the genomic rearrangements coincide with gaps or breakpoints in the assembly, then they would not be detected.

A previous study reported large genomic deletions in about 5% of the examined *Xanthomonas* strains, including Xff 4834-R, resulting in loss of flagellar motility (Darrasse et al., 2013b). Although, none of the genomes sequenced in the present study displayed this deletion, there were several other strain-specific multi-kilobase deletions (see Figure 1) suggesting that this is a relatively common phenomenon among xanthomonads.

## Discussion

In the present study, we sequenced the genomes of 26 strains of the causative agents of CBB, whose times and places of isolation spanned several decades and several continents. This resource adds to the already published genome sequences of *Xff* 4834-R and CFBP 4884 (Darrasse et al., 2013b; Indiana et al., 2014) with a further 13 sequenced genomes. We also present the first genome sequences for *Xap*, including 9 strains belonging to a previously described lineage known as GL 1. These 9 sequenced GL 1 strains include 8 from common bean and one from Lima bean. We also sequenced a further four strains from lablab bean.

Our data reveal genetic sub-lineages within *Xff* and within *Xap* GL 1, each having a widely dispersed geographical distribution. The availability of these genome sequence data will be a useful source of genetic variation for use in developing molecular markers for distinguishing individual sub-lineages or genotypes and thus aiding the study of routes of pathogen spread (Vinatzer et al., 2014; Goss, 2015). We observed considerable intra-lineage variation with respect to gene content as well as single-nucleotide variations (Figures 4–7).

Whole-genome sequencing revealed the repertoires of predicted T3SS effectors. Our results (Figure 9) were consistent with a previous survey of effector genes (Hajri et al., 2009) except for two apparent discrepancies. First, we find no evidence for presence of *avrRxo1* (*xopAJ*) in the genomes of *Xap* nor *Xff* though Hajri and colleagues found this gene in *Xap* GL 1. Second, genome-wide sequencing sequencing was able to distinguish between *xopF1* and *xopF2*. We find *xopF1* in both *Xff* and *Xap* GL 1 but find *xopF2* only in *Xff*. Hajri reported presence of *xopF2* in both *Xff* and *Xap* GL1; this might be explained by cross-hybrisisation of *xopF1* with the *xopF2* probes.

Arguably the most surprising finding to arise from the present study is the observation that Xff and Xap GL 1 share 100% identical alleles at dozens of loci even though on average most loci share only about 96% identity. This phenomenon is apparent from the bimodal distributions of sequence identities in Figure 11. This phenomenon is apparently restricted to sharing between GL 1 and Xff; no such bimodal distribution is seen between GL 1 and the lablab-associated strain not between GL 1 and *X. fuscans* subsp. *aurantifolii* (which is closely related to Xff). Furthermore, many of the alleles sharing 100% identity between GL 1 and *Xff* show significantly less identity between *Xap* GL 1 and *X. axonopodis* pv. *manihotis*, despite the close phylogenetic relationship between these last two. On the other hand, these shared sequences are more similar to sequences from *X. fuscans* subsp. *aurantifolii* than to sequence from *X. axonopodis* pv. *manihotis*, suggesting that they were acquired by *Xap* GL 1 from *Xff* rather than *vice versa*. Examples are listed in Table 2. It remains to be tested whether these alleles are adaptive for survival on a common ecological niche.

### Conflict of interest statement

The authors declare that the research was conducted in the absence of any commercial or financial relationships that could be construed as a potential conflict of interest.

## Footnotes

^1^Andrews, S. FastQC A Quality Control tool for High Throughput Sequence Data. Available online at: http://www.bioinformatics.babraham.ac.uk/projects/fastqc/ [Accessed May 13, 2015].
