# Supplementary material for: Genome sequencing reveals a new lineage associated with lablab bean and genetic exchange between Xanthomonas axonopodis pv. phaseoli and Xanthomonas fuscans subsp. fuscans
Source: Front Microbiol. 2015 Oct 7;6:1080. doi: 10.3389/fmicb.2015.01080 (PMC4595841; doi:10.3389/fmicb.2015.01080)
Supplement: Supplementary file 2 [file SupplementaryFigures.PDF]

Frontiers in Microbiology

Genome sequencing reveals a new lineage associated with lablab bean and genetic exchange between *Xanthomonas axonopodis* pv. *phaseoli* and *Xanthomonas fuscans* subsp. fuscans

Valente Aritua[1], James Harrison[2], Melanie Sapp[3], Robin Buruchara[4], Julian Smith[3], David J. Studholme[2]*

[1]International Center for Tropical Agriculture, Kampala, Uganda

[2]Biosciences, University of Exeter, Exeter, United Kingdom

[3]Fera, York, United Kingdom

[4]CIAT Africa Regional Office, International Center for Tropical Agriculture, Nairobi, Kenya

*** Correspondence:** David J. Studholme, Biosciences, University of Exeter, Stocker Road, Exeter, EX4 4QD, United Kingdom.
D.J.Studholme@exeter.ac.uk

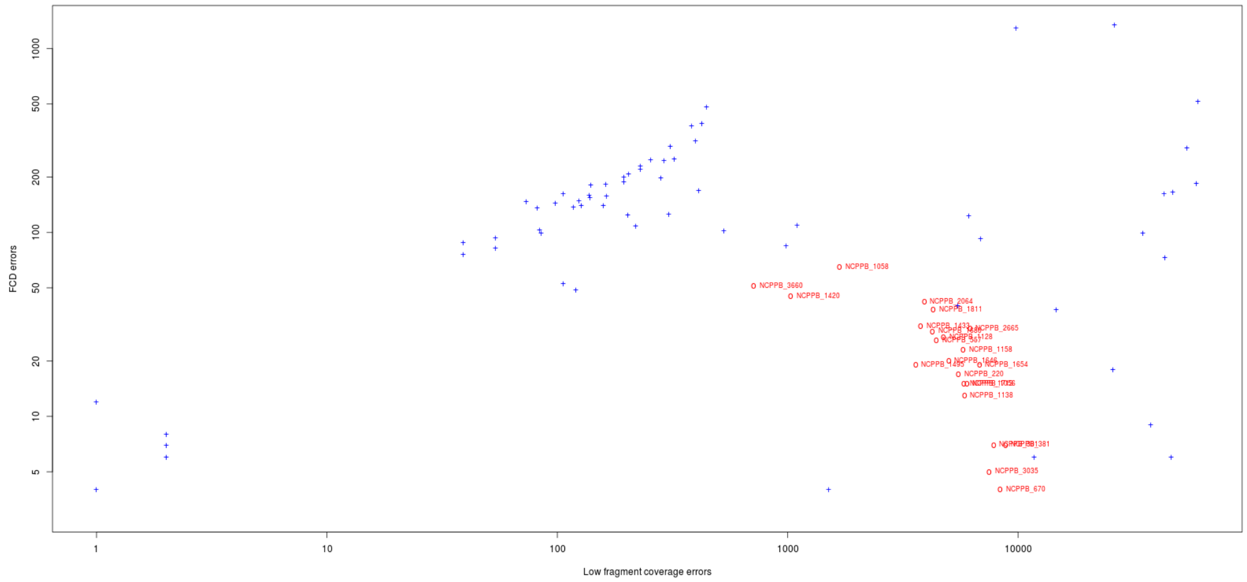

Figure S1 Comparison of assembly accuracy. To assess the accuracy of the newly assembled genome sequences, without a ground-truth reference genome sequence against which to compare, we used the REAPR tool (Hunt et al., 2013). REAPR detects two classes of potential errors in assemblies: Fragment coverage distribution (FCD) errors and low fragment coverage errors. We ran REAPR against each of the assemblies generated in the present study and also against each of the assemblies of *X. axonopodis* pv. *manihotis* described in a previously published study (Bart et al., 2012). A perfect error-free assembly would fall in the bottom left corner of the plot whereas a poor error-rich sequence would fall in the top right.

Red circles (**o**): Genome sequence assemblies from the present study.

Blue crosses (+): Genome assemblies from previously published study (Bart et al., 2012).

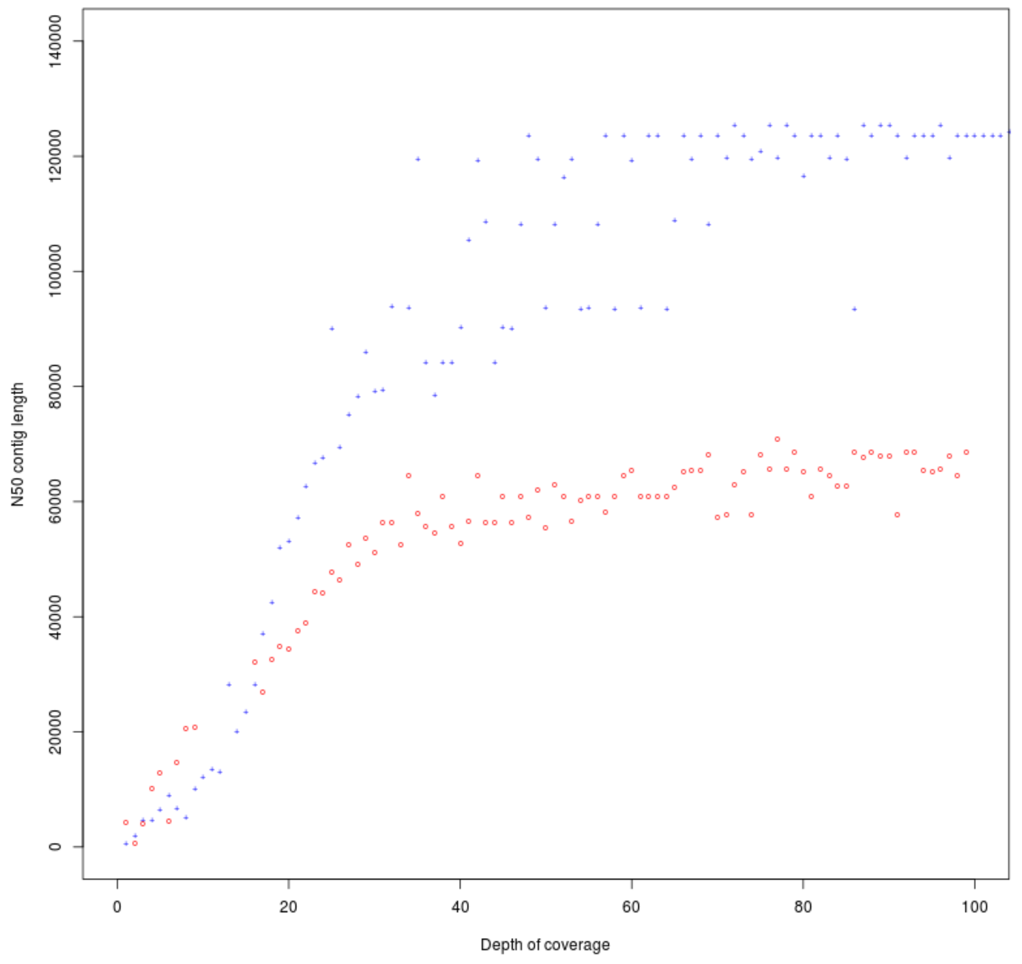

Figure S2 Effect of depth of coverage on the contiguity of genome assembly. To assess the effect of depth of read coverage on assembly contiguity, we subsampled reads from the datasets for strains NCPPB 2064 and NCPPB 1058 to give a range of depths from 1 to 100 x. Depth of coverage was calculated by dividing the total number of nucleotides in the input sequence reads by 4,700 (i.e. assuming that the genome size is 4.7 Mb). Each subsample of reads was assembled with SPAdes using the same protocol as for the assemblies described in the main text. The N_{50} contig length was calculated for each assembly using Quast (Gurevich et al., 2013).

Red circles (**o**): Genome sequence assemblies of *Xff* NCPPB 1058.

Blue crosses (+): Genome assemblies of *Xap* NCPPB 2064.

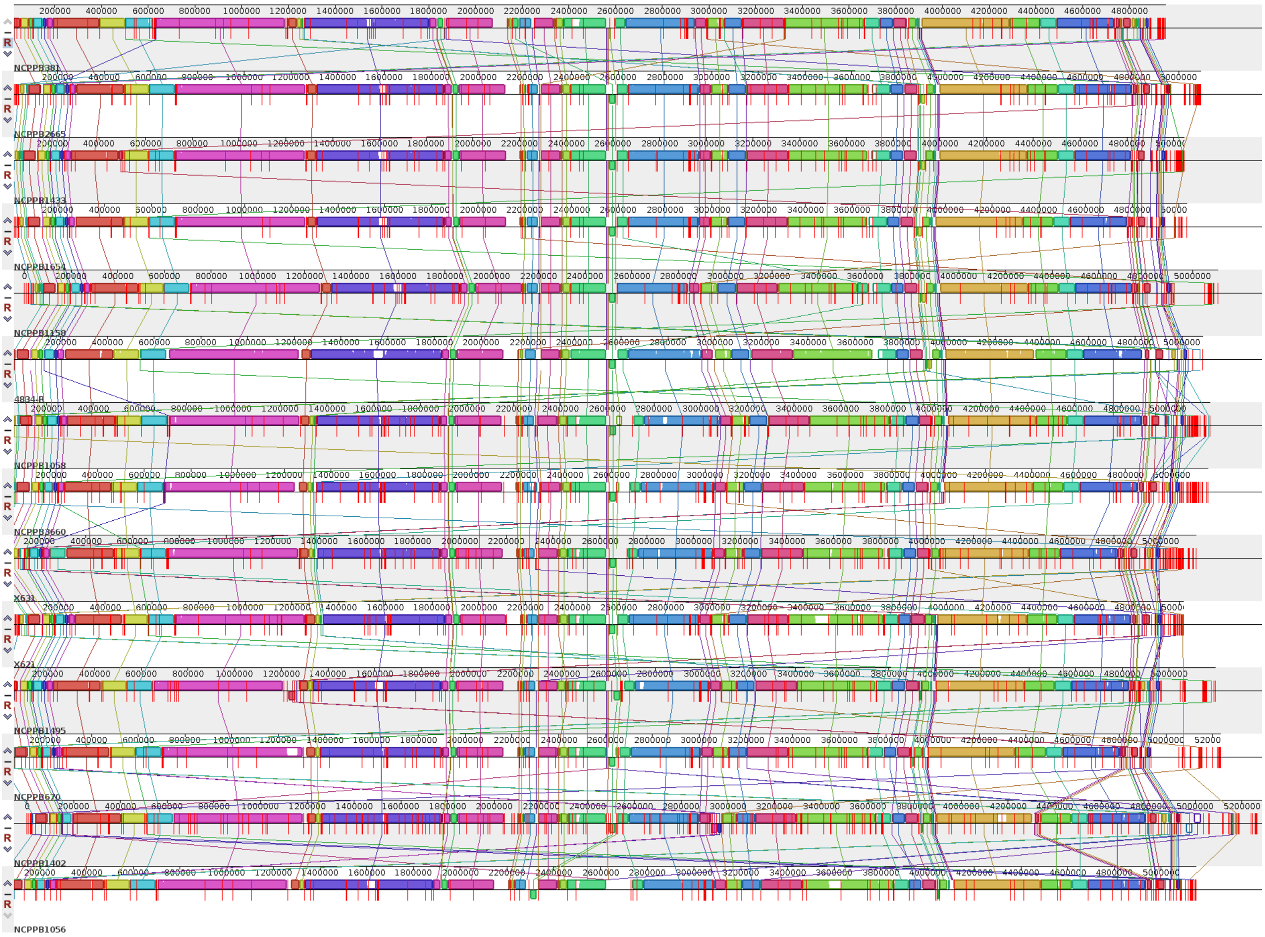

Figure S3 Whole-genome alignments of *X. fuscans* subsp. *fuscans*. The genome assemblies of each sequenced isolate were aligned, and using Mauve (Darling et al., 2004, 2010; Rissman et al., 2009).

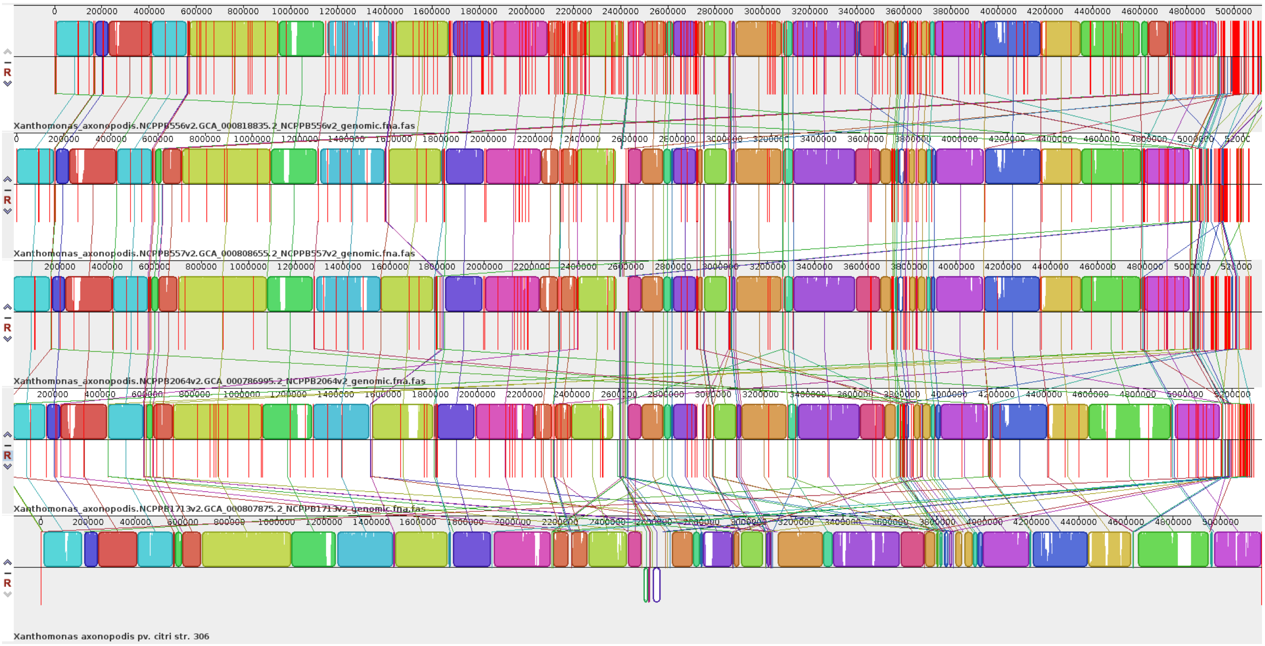

Figure S4 Whole-genome alignments of *X. phaseoli* pv. *phaseoli* lablab-associated isolates. The genome assemblies of each sequenced isolate were aligned, and using Mauve (Darling et al., 2004, 2010; Rissman et al., 2009).

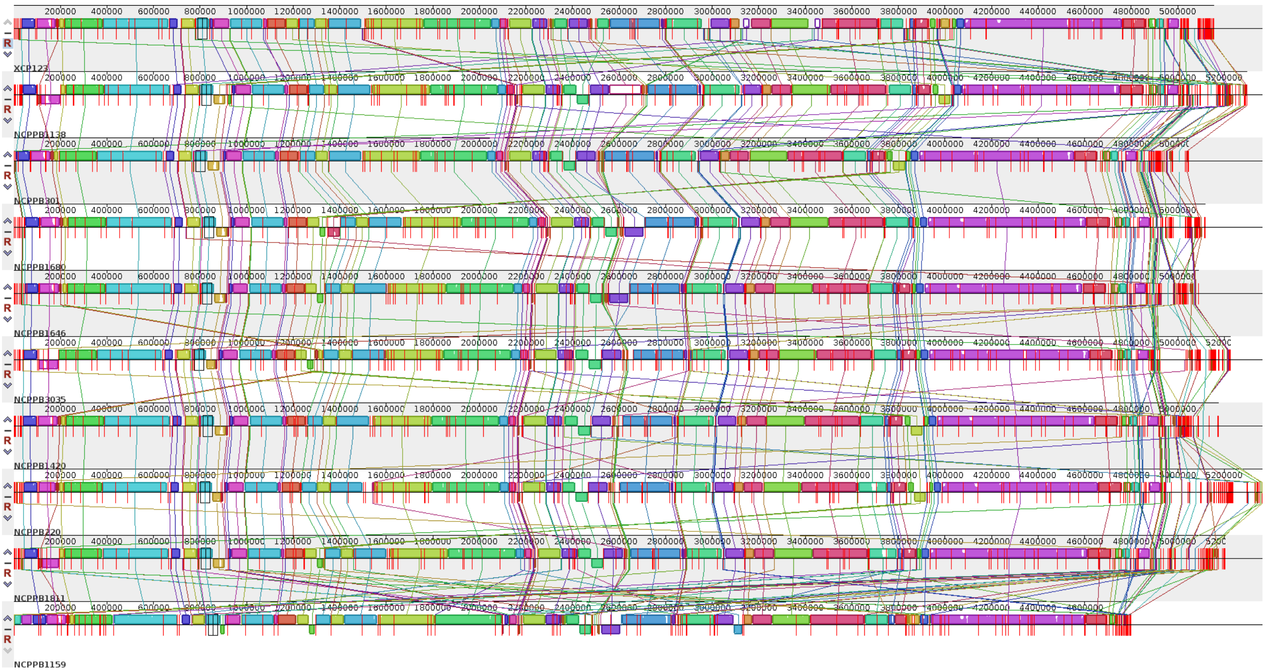

Figure S5 Whole-genome alignments of *X. phaseoli* pv. *phaseoli* genetic lineage 1 (GL1). The genome assemblies of each sequenced isolate were aligned, and using Mauve (Darling et al., 2004, 2010; Rissman et al., 2009). The genome assembly of *X. axonopodis* pv. *manihotis* NCPPB 1159 (Bart et al., 2012) is also included for comparison.

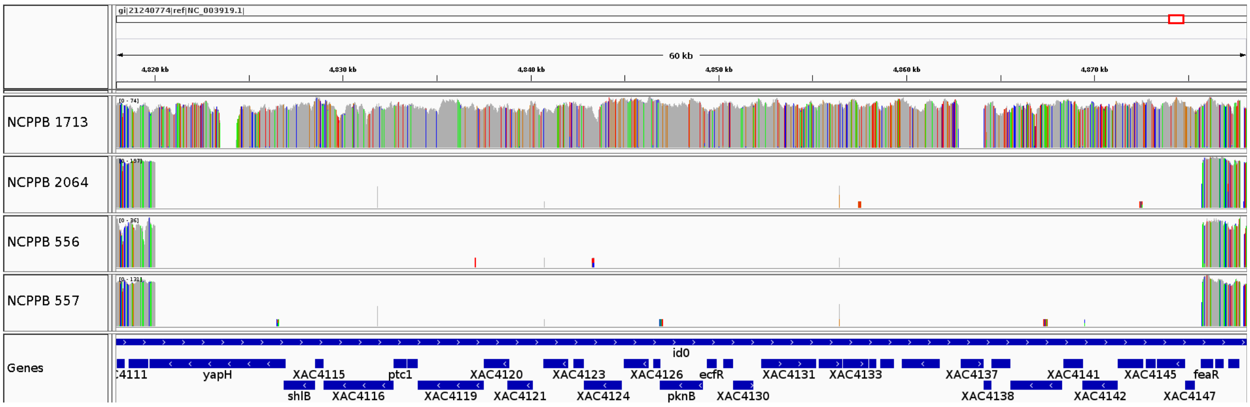

Figure S6 A 60-kbp genomic deletion found in Sudanese lablab-associated isolates NCPPB 2064, NCPPB 556 and NCPPB 557 but not in Zimbabwean isolate NCPPB 1713. The MiSeq sequence reads were aligned against the reference genome sequence of *X. axonopodis* pv. *citri* 306 (da Silva et al., 2002) using BWA-MEM (Li, 2013, 2014). The depth of coverage plots are visualised using IGV (Thorvaldsdóttir et al., 2013).

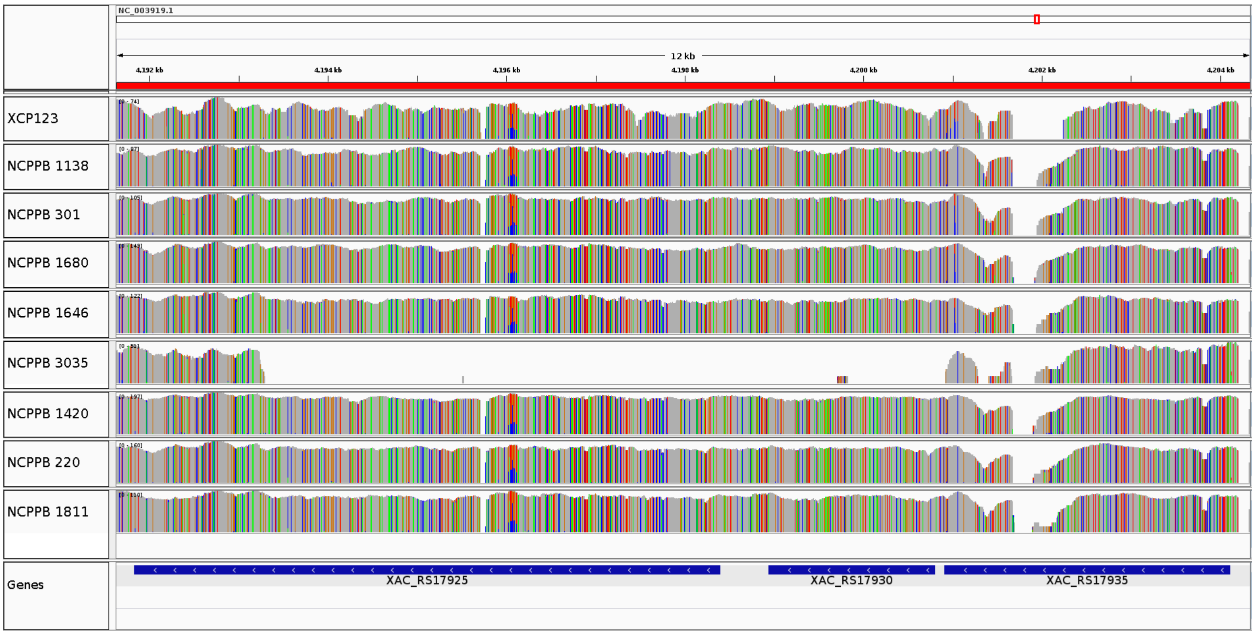

Figure S7 A 8-kbp genomic deletion found in *Xap* NCPPB 3035 but not in other sequenced Xap GL1 isolates. The MiSeq sequence reads were aligned against the reference genome sequence of *X. axonopodis* pv. *citri* 306 (da Silva et al., 2002) using BWA-MEM (Li, 2013, 2014). The depth of coverage plots are visualised using IGV (Thorvaldsdóttir et al., 2013).

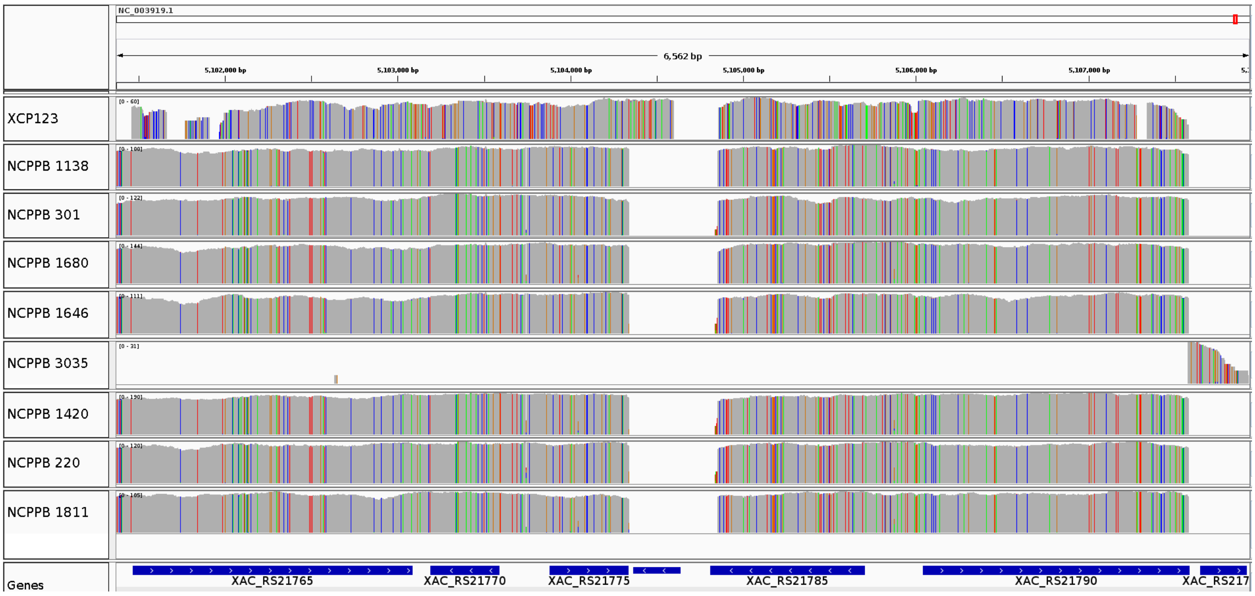

Figure S8 A 6-kbp genomic deletion found in *Xap* NCPPB 3035 but not in other sequenced Xap GL1 isolates. The MiSeq sequence reads were aligned against the reference genome sequence of *X. axonopodis* pv. *citri* 306 (da Silva et al., 2002) using BWA-MEM (Li, 2013, 2014). The depth of coverage plots are visualised using IGV (Thorvaldsdóttir et al., 2013).

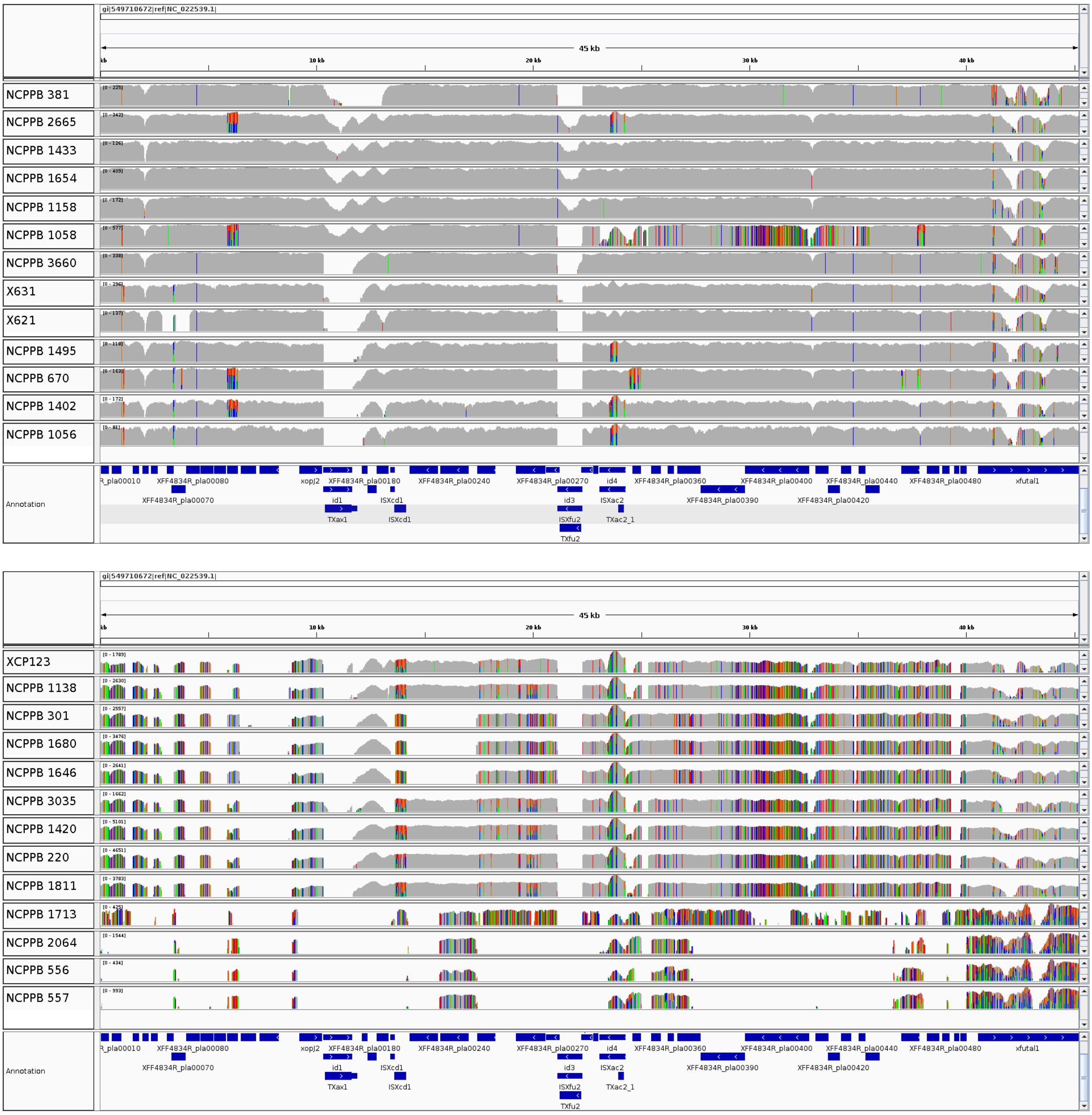

Figure S9 Conservation of *Xff* plasmid pla in the *Xff* and *Xap* isolates sequenced in the present study. The MiSeq sequence reads were aligned against the reference genome sequence of *Xff* 4834-R (Darrasse et al., 2013) using BWA-MEM (Li, 2013, 2014). The depth of coverage plots are visualised using IGV (Thorvaldsdóttir et al., 2013).

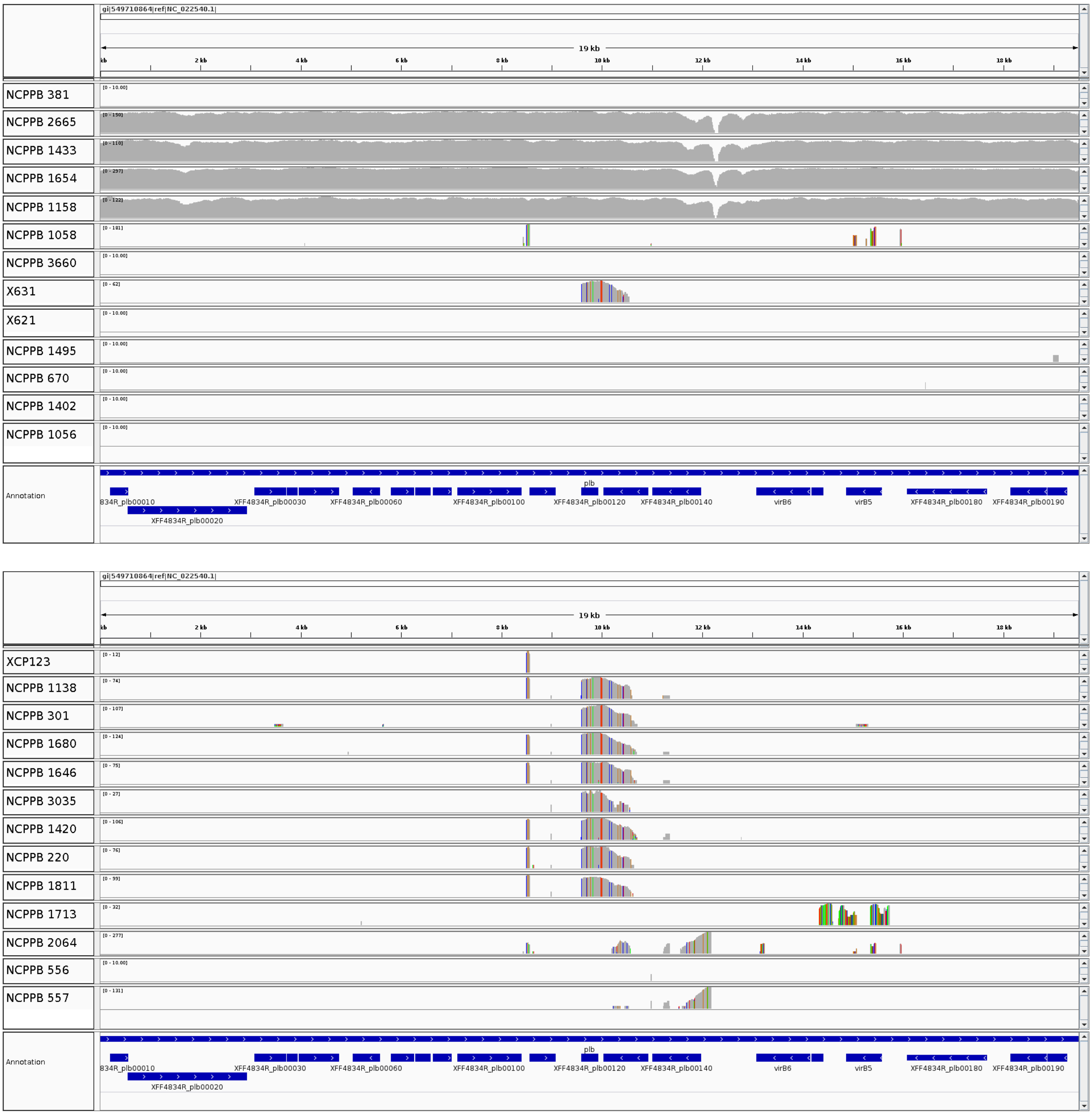

Figure S10 Conservation of *Xff* plasmid plb in the *Xff* and *Xap* isolates sequenced in the present study. The MiSeq sequence reads were aligned against the reference genome sequence of *Xff* 4834-R (Darrasse et al., 2013) using BWA-MEM (Li, 2013, 2014). The depth of coverage plots are visualised using IGV (Thorvaldsdóttir et al., 2013).

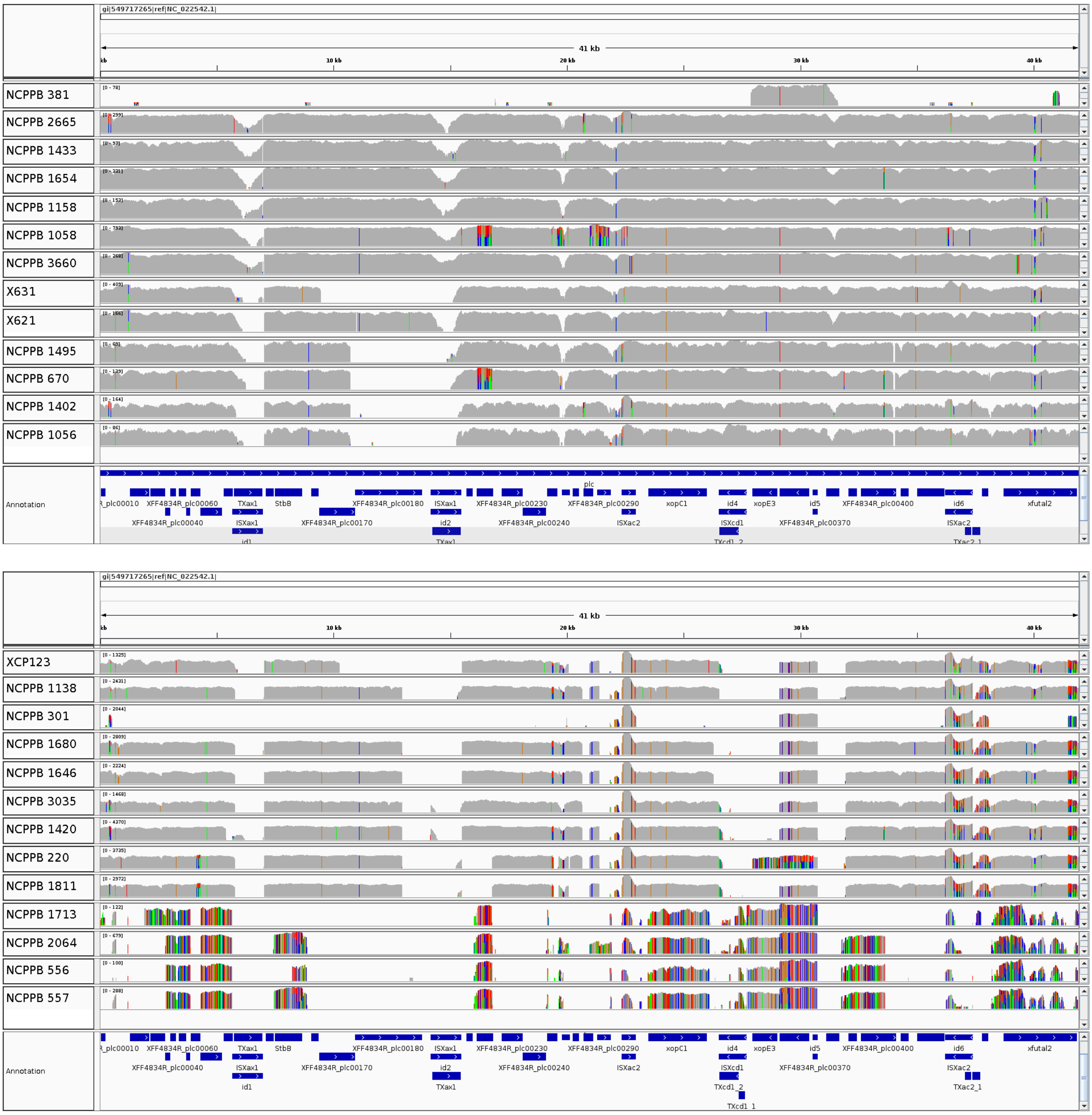

Figure S10 Conservation of *Xff* plasmid plc in the *Xff* and *Xap* isolates sequenced in the present study. The MiSeq sequence reads were aligned against the reference genome sequence of *Xff* 4834-R (Darrasse et al., 2013) using BWA-MEM (Li, 2013, 2014). The depth of coverage plots are visualised using IGV (Thorvaldsdóttir et al., 2013).

References

Bart, R., Cohn, M., Kassen, A., McCallum, E. J., Shybut, M., Petriello, A., Krasileva, K., Dahlbeck, D., Medina, C., Alicai, T., et al. (2012). High-throughput genomic sequencing of cassava bacterial blight strains identifies conserved effectors to target for durable resistance. *Proc. Natl. Acad. Sci. U. S. A.* 109, E1972–9. doi:10.1073/pnas.1208003109.

Darling, A. C. E., Mau, B., Blattner, F. R., and Perna, N. T. (2004). Mauve: Multiple alignment of conserved genomic sequence with rearrangements. *Genome Res.* 14, 1394–1403. doi:10.1101/gr.2289704.

Darling, A. C. E., Mau, B., and Perna, N. T. (2010). progressiveMauve: Multiple Genome Alignment with Gene Gain, Loss and Rearrangement. *PLoS One* 5, e11147. doi:10.1371/journal.pone.0011147.

Darrasse, A., Carrère, S., Barbe, V., Boureau, T., Arrieta-Ortiz, M. L., Bonneau, S., Briand, M., Brin, C., Cociancich, S., Durand, K., et al. (2013). Genome sequence of Xanthomonas fuscans subsp. fuscans strain 4834-R reveals that flagellar motility is not a general feature of xanthomonads. *BMC Genomics* 14, 761. doi:10.1186/1471-2164-14-761.

Gurevich, A., Saveliev, V., Vyahhi, N., and Tesler, G. (2013). QUAST: quality assessment tool for genome assemblies. *Bioinformatics* 29, 1072–5. doi:10.1093/bioinformatics/btt086.

Hunt, M., Kikuchi, T., Sanders, M., Newbold, C., Berriman, M., and Otto, T. D. (2013). REAPR: a universal tool for genome assembly evaluation. *Genome Biol.* 14, R47. doi:10.1186/gb-2013-14-5-r47.

Li, H. (2013). Aligning sequence reads, clone sequences and assembly contigs with BWA-MEM. 3. Available at: http://arxiv.org/abs/1303.3997 [Accessed July 20, 2014].

Li, H. (2014). Toward better understanding of artifacts in variant calling from high-coverage samples. *Bioinformatics* 30, 1–9. doi:10.1093/bioinformatics/btu356.

Rissman, A. I., Mau, B., Biehl, B. S., Darling, A. E., Glasner, J. D., and Perna, N. T. (2009). Reordering contigs of draft genomes using the Mauve aligner. *Bioinformatics* 25, 2071–3. doi:10.1093/bioinformatics/btp356.

Da Silva, A. C. R., Ferro, J. A., Reinach, F. C., Farah, C. S., Furlan, L. R., Quaggio, R. B., Monteiro-Vitorello, C. B., Van Sluys, M. A., Almeida, N. F., Alves, L. M. C., et al. (2002). Comparison of the genomes of two Xanthomonas pathogens with differing host specificities. *Nature* 417, 459–63. doi:10.1038/417459a.

Thorvaldsdóttir, H., Robinson, J. T., and Mesirov, J. P. (2013). Integrative Genomics Viewer (IGV): high-performance genomics data visualization and exploration. *Briefings Bioinforma.* 14 , 178–192. doi:10.1093/bib/bbs017.
